# The Capacity of High-Grade Serous Ovarian Cancer Cells to Form Multicellular Structures Spontaneously along Disease Progression Correlates with Their Orthotopic Tumorigenicity in Immunosuppressed Mice

**DOI:** 10.3390/cancers12030699

**Published:** 2020-03-16

**Authors:** Alicia Goyeneche, Michael-Anthony Lisio, Lili Fu, Rekha Srinivasan, Juan Valdez Capuccino, Zu-hua Gao, Carlos Telleria

**Affiliations:** 1 Experimental Pathology Unit, Department of Pathology, Faculty of Medicine, McGill University, Montreal, QC H3A 2B4, Canada; michael-anthony.lisio@mail.mcgill.ca (M.-A.L.); Lili.Fu@muhc.mcgill.ca (L.F.); zu-hua.gao@mcgill.ca (Z.-h.G.); carlos.telleria@mcgill.ca (C.T.); 2 Division of Basic Biomedical Sciences, Sanford School of Medicine, The University of South Dakota, Vermillion, SD 57069, USA; rekha.srinivasan@usd.edu (R.S.); jm.valdez88@gmail.com (J.V.C.)

**Keywords:** high-grade serous ovarian cancer, spheroids, aggregates, multicellular structures, peritoneal carcinomatosis, cancer biology

## Abstract

Many studies have examined the biology, genetics, and chemotherapeutic response of ovarian cancer’s solid component; its liquid facet, however, remains critically underinvestigated. Floating within peritoneal effusions known as ascites, ovarian cancer cells form multicellular structures, creating a cancer niche in suspension. This study explores the pathobiology of spontaneously formed, multicellular, ovarian cancer structures derived from serous ovarian cancer cells isolated along disease evolution. It also tests their capacity to cause peritoneal disease in immunosuppressed mice. Results stem from an analysis of cell lines representing the most frequently diagnosed ovarian cancer histotype (high-grade serous ovarian cancer), derived from ascites of the same patient at distinct stages of disease progression. When cultured under adherent conditions, in addition to forming cellular monolayers, the cultures developed areas in which the cells grew upwards, forming densely packed multilayers that ultimately detached from the bottom of the plates and lived as free-floating, multicellular structures. The capacity to form foci and to develop multicellular structures was proportional to disease progression at the time of ascites extraction. Self-assembled in culture, these structures varied in size, were either compact or hollow, irregular, or spheroidal, and exhibited replicative capacity and an epithelial nature. Furthermore, they fully recreated ovarian cancer disease in immunosuppressed mice: accumulation of malignant ascites and pleural effusions; formation of discrete, solid, macroscopic, peritoneal tumors; and microscopic growths in abdominal organs. They also reproduced the histopathological features characteristic of high-grade serous ovarian cancer when diagnosed in patients. The following results encourage the development of therapeutic interventions to interrupt the formation and/or survival of multicellular structures that constitute a floating niche in the peritoneal fluid, which in turn halts disease progression and prevents recurrence.

## 1. Introduction

Ovarian cancer is a deadly disease with unique features when compared to other solid tumors. For instance, in ovarian cancer, dissemination through the vasculature is rare and a very late manifestation of the disease. Instead, ovarian cancer cells are prone to spreading by direct extension into adjacent tissues or by detaching from the primary tumor directly into the abdominal cavity, where they seed the mesothelium of the omentum, diaphragm, bowel serosa, and the entire peritoneum [1,2,3]. Despite aggressive surgical resection of widely disseminated visible and palpable tumors, followed by chemotherapy, most patients with advanced ovarian cancer at presentation, which accounts for 70% of cases, will relapse and succumb to the disease [4]. Widespread visceral and intestinal wall tumors with adhesions formed between the loops of the bowel cause intestinal obstruction, prevent normal nutrition, and become a primary cause of death [5]. 

The pathogenicity of the disease within the abdominal cavity is driven by solid components as well as cellular effusions within the peritoneal cavity (ascites) found in most grade III International Federation of Gynecology and Obstetrics (FIGO) or pleural cavity (pleural effusions) found in grade IV FIGO; colonization of the pleural cavity is a marker of distant dissemination in this disease [4,6,7]. A large number of investigations have focused on the understanding of the biology, genetics, and response to chemotherapeutic agents of the solid component of the disease. In contrast, a less understood aspect of ovarian cancer is the role of ovarian cancer cells arranged as multicellular structures that form a cancer niche in suspension. Malignant cells that spread outside their primary site of origin are found floating within the peritoneal cavity, a tendency that is confirmed by peritoneal washes being positive in ~80% of ovarian cancer diagnoses, which correlates strongly with elevated morbidity and mortality, regardless of disease stage [8]. Moreover, ~65% of patients diagnosed at advanced presentation have symptoms of ascites accumulation [6,7]. Similar symptoms are developed as a consequence of ascites accumulation at recurrence [9]. Altogether, clinical evidence strongly suggests that the liquid component of ovarian cancer is an active pathogenic manifestation of the disease. This liquid disease component is more abundantly present in patients diagnosed with high-grade serous ovarian cancer (HGSOC), when compared to the other less frequently diagnosed ovarian cancer histotypes [10]. 

This work explores the biology of HGSOC cells [11] isolated along disease evolution, examines their capacity to form multicellular structures under culture conditions, and assesses their ability to cause peritoneal disease in immunosuppressed mice. Results show that multicellular ovarian cancer structures formed spontaneously in culture vary in size, are either compact or hollow, irregular, or spheroidal, and have replicative capacity as well as an epithelial nature. Furthermore, this investigation demonstrates that the capacity of the cells, obtained along disease progression, to form multicellular structures spontaneously, is directly related to the in vivo tumorigenicity when injected into the abdominal cavity of immunosuppressed mice. This paper shows that spontaneously formed multicellular structures are sufficient to fully recreate the disease, including an accumulation of malignant ascites and pleural effusions, and the formation of solid, discrete, macroscopic tumors and multiple microscopic growths in abdominal organs. 

## 2. Results

### 2.1. Serous Ovarian Cancer Cells of Advanced Disease Self-Assemble into Multicellular Structures in Suspension Despite Being Cultured in Anchorage-Prone Conditions

**Figure 1:**
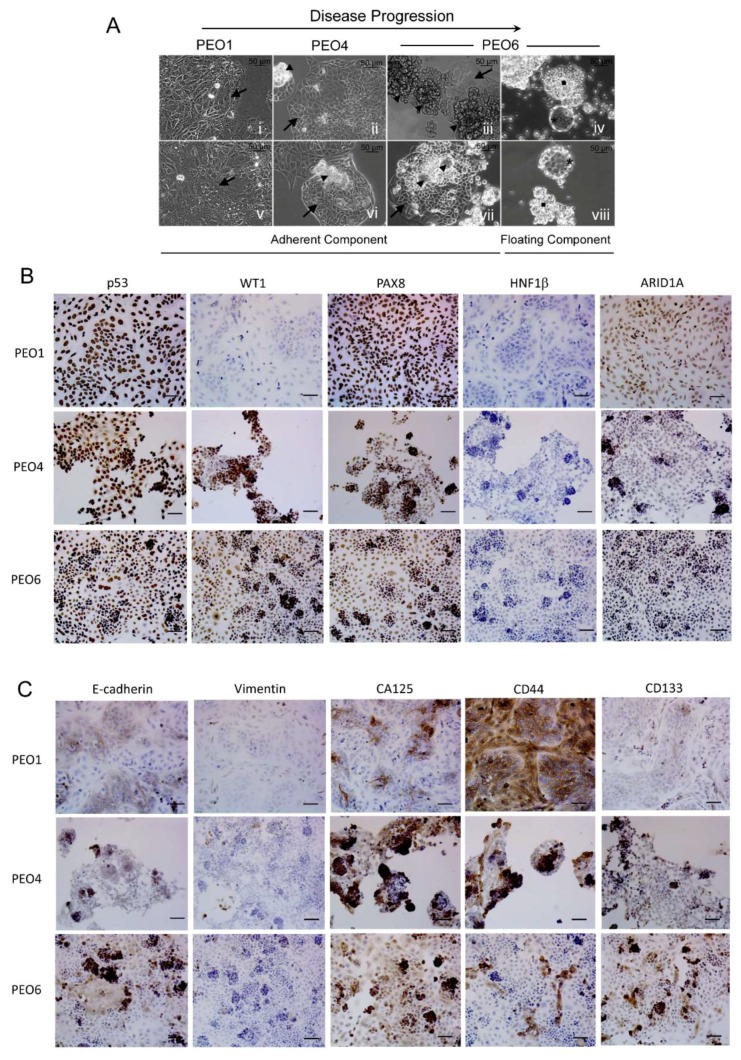
(**A**) PEO1, PEO4, and PEO6 cells were cultured for 10 days in media with 10% serum, including insulin and antibiotics. *Arrows*: adherent cells. *Arrowheads*: 3D foci. *Squares*: irregular multicellular structures. *Asterisks*: spheroidal multicellular structures. (**B**) Expression of biomarkers of HGSOC in cultured PEO1/4/6 cells. (**C**) Expression of markers of epithelial-mesenchymal transition and of stem cell plasticity in PEO1/4/6 cells. Scale bars = 50 μm.

In this work, we set out to investigate the orthometastatic capacity of a series of high-grade serous cell lines derived from the same patient along disease evolution. We used three poorly differentiated serous ovarian cancer cell lines developed longitudinally from ascites of the same patient following initial chemotherapy (PEO1), when clinically chemoresistant (PEO4), and prior to death (PEO6) [12,13]. We cultured these serous ovarian cancer cells under standard conditions for adherent cells, i.e., an anchorage-prone setting and differentiation medium containing fetal bovine serum. Under these conditions, some of these cells developed tridimensional (3D) foci that remained attached to a flat-adherent phenotypic component (Figure 1A). The capacity of the cells to form foci was proportional to the progression of the disease in the patient from whom the cells were obtained. PEO1 cells do not form apparent foci (Figure 1A [i,v]), PEO4 form some scattered foci (Figure 1A [ii,vi]), and PEO6 form abundant foci in culture (Figure 1A [iii,vii]). 

The capacity to form foci in culture by the cell lines was also related to their capacity to form non-adherent multicellular structures. These multicellular structures found in suspension were at least of two kinds: irregular or grape-like and more organized, or spheroidal, as clearly shown for PEO6 cells (Figure 1A [iv,viii]). Comparable features were also observed, though very scarcely, in the PEO14 and PEO23 cell pair, obtained from the same patient before chemotherapy (PEO14), and at relapse following chemotherapy (PEO23). While both cell lines formed foci in culture, only PEO23, obtained at more advanced disease, developed some floating irregular or spheroidal multicellular structures (Figure S1). The results illustrate the capacity of serous ovarian cancer cells from advanced disease to spontaneously form multicellular structures under adherent conditions and without any additional manipulation to promote cellular gathering.

### 2.2. Expression of Biomarkers in High-Grade Serous Ovarian Cancer Cells along Disease Progression

We next characterized the cell lines for their capacity to express biomarkers of HGSOC, markers of epithelial-mesenchymal transition, and stem cell markers. Figure 1B shows that PEO1, PEO4, and PEO6 cells accumulate p53, which denotes the mutant nature of the tumor suppressor protein [13]. The high-grade serous marker WT1 was barely expressed in PEO1 cells, but instead, it was highly expressed in PEO4 and PEO6 cells, representing more advanced disease. WT1 seems to reach its maximal expression when the cells form aggregates and when they float as multicellular structures (Figure S2). The PEO1/4/6 series also expresses PAX8 as a biomarker of HGSOC [14,15,16]. As expected, the cells stained negative for the clear-cell ovarian cancer marker HNF1β [17]. All cell lines show positivity for ARID1A, which is a biomarker found mutant and undetectable with high frequency in endometroid and clear-cell adenocarcinomas [18], but it is often expressed in HGSOC [17]. In Figure 1C, we also show the expression of the epithelial marker E-cadherin and mesenchymal cell marker vimentin in all cell lines [19,20]. E-cadherin is expressed in pockets intertwined with vimentin positivity in PEO1 cells. It is clear that when the disease advances and begins to form clusters of cellular aggregates, such cells are highly positive for E-cadherin losing vimentin expression (the cases of PEO4 and PEO6). The epithelial ovarian cancer biomarker CA125 [21] is also expressed, first in pockets in PEO1 cultures, but it becomes more abundant when the cells form cellular aggregates in the cases of PEO4 and PEO6 cells. Additionally, the stem cell marker CD44 [20] is highly expressed in PEO1 cells, while it is expressed only in pockets in PEO4 and PEO6 cells. Finally, the stem cell marker CD133 [22] has low expression in PEO1 cells, increasing instead in PEO4 and PEO6 cells. We confirmed in more detail the expression of CD133 in the plasma membrane of non-adherent PEO6 cells by immunofluorescence (Figure S3). In summary, the expression of biomarkers in these longitudinally obtained HGSOC cell lines depicts how the disease adapts along its progression following the different rounds of chemotherapy the patient underwent in between their generation. Along disease progression, cells clearly express markers of HGSOC and also display phenotypic plasticity in terms of the expression of epithelial/mesenchymal as well as stem cell markers. 

### 2.3. Both Flat Adherent Cells and Cells Within Multicellular Structures Have Proliferative Capacity and Express E-Cadherin

**Figure 2:**
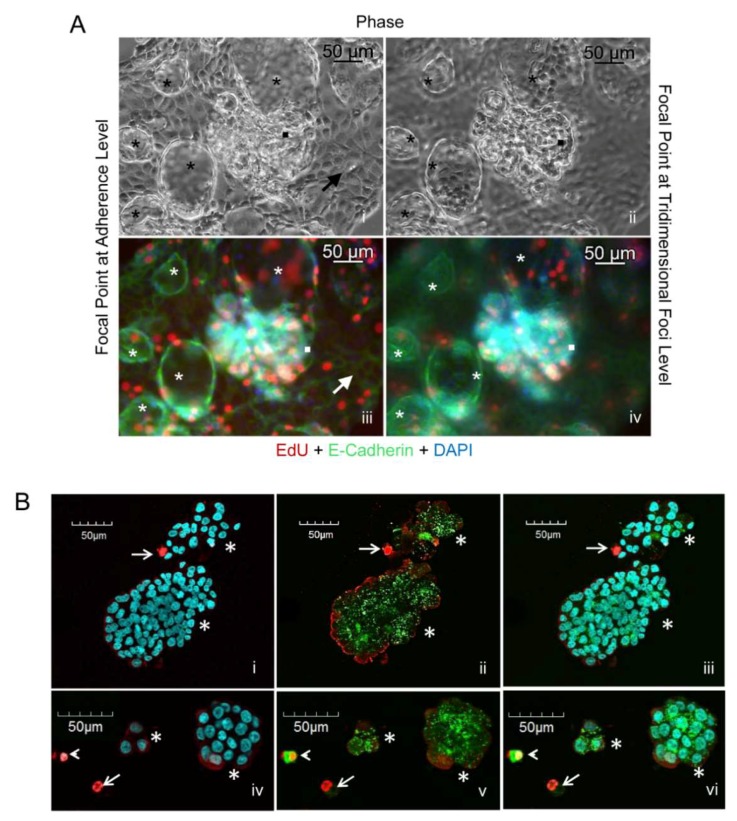
(**A**) Phase contrast imaging at focal [i] and 3D planes [ii] of PEO6 cells displaying a cellular monolayer (*arrow*), 3D irregular multicellular structures (*squares*), and spheroidal multicellular structures (*asterisks*). EdU incorporation (red fluorescence) and E-cadherin expression (green fluorescence) are shown at the focal plane [iii] and at the 3D plane [iv]. Blue, DAPI, depicting nuclei. (**B**) Live/dead^®^ viability/cytotoxicity assay of PEO6 cells arranged as multicellular structures as stained with DAPI [i, iv], calcein AM and EthD-1 [ii, v], and their overlay [iii, vi]. *Asterisks* denote multicellular structures mostly alive as assessed by their green calcein AM staining. *Arrowheads* denote dying cells (yellow), whereas *arrows* identify dead cells (red).

We further studied the capacity of PEO6 cells, either adherent or gathered as multicellular structures, to proliferate and to express the epithelial marker E-cadherin. PEO6 cultures containing adherent cells, and multicellular irregular structures or more organized spheroidal structures (black asterisks in Figure 2A [i, ii]), were exposed to the BrdU mimetic EdU to assess their capacity to synthesize DNA. EdU positive cells were observed in both flat and 3D culture components; cells growing in monolayer, shown at the focal point, or multicellular structures, shown at the 3D plane (Figure 2A [iii,iv]), have proliferative capacities. This figure also shows that the cell–cell adhesion protein E-cadherin is expressed in cells growing in monolayer, and that such expression is not lost when the cells gather as multicellular structures, thus denoting their epithelial nature. 

### 2.4. Free-Floating Multicellular Structures Are Viable Entities

We next assessed the viability of the non-adherent multicellular structures. We subjected a preparation of multicellular structures floating in suspension to a two-fluorochrome viability/cytotoxicity assay. Results in Figure 2B show that the non-adherent components, when aggregated, are mostly composed of live cells. 

### 2.5. The Free-Floating Multicellular Structures Generated Spontaneously from a Culture of PEO6 Cells Display a Coral-Like or Irregular Phenotype Together with More Organized Spheroidal Arrangement 

**Figure 3:**
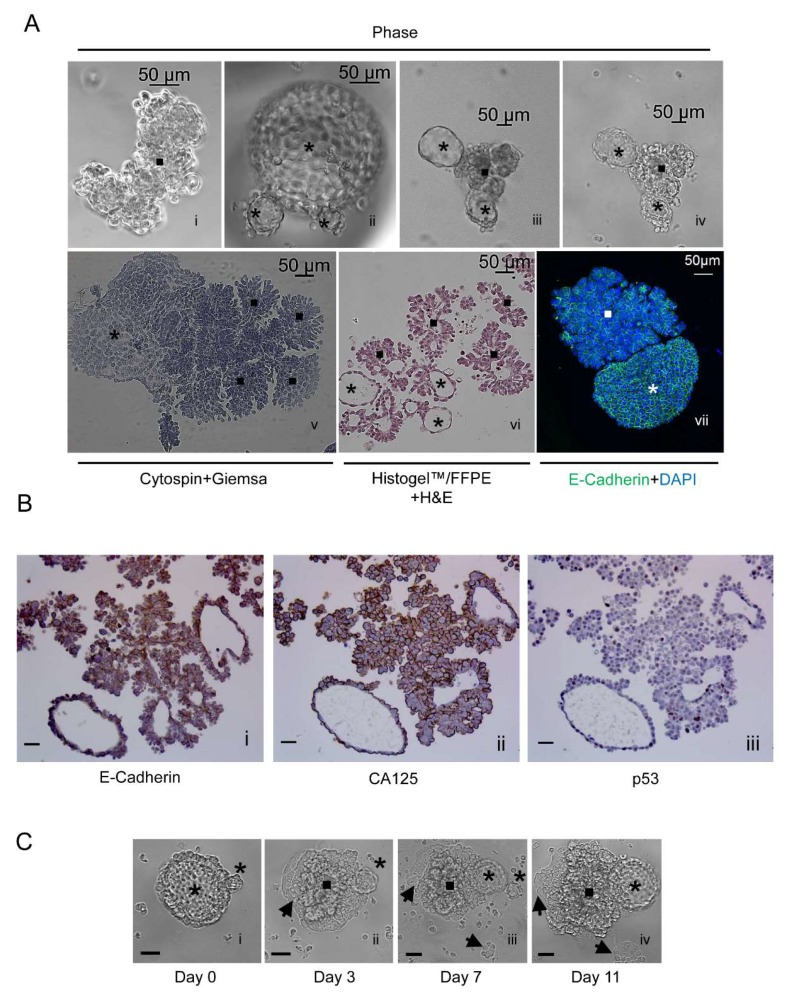
(**A**) [i–iv] Phase-contrast images of irregular and spheroidal PEO6 multicellular structures; [v] cytospin and Giemsa staining; [vi] H&E staining of a 5 μm section of multicellular structures formalin-fixed and paraffin-embedded (FFPE), and solidified with Histogel^TM^; [vii] cytospin and immunofluorescence of E-cadherin (green) and nuclear staining with DAPI (blue); *asterisks*, spheroidal multicellular structures; *squares*, irregular multicellular structures. (**B**) Non-adherent multicellular structures were fixed, solidified in Histogel^TM^, embedded in paraffin, and subjected to immunocytochemical staining for E-Cadherin [i], CA125 [ii], or p53 [iii]. (**C**) A spheroidal multicellular structure is observed using phase-contrast microscopy when floating [i], after attachment [ii], and after further development in culture [iii], giving rise to newly formed multicellular structures [iv]. *Asterisk*, spheroidal multicellular structures; *square*, multicellular foci; *arrowheads*, cellular structures that remain adherent to the plastic surface. Scale bars in B and C, 50 μm.

Floating PEO6 multicellular structures denote the presence of irregular structures associated with highly organized spheroids within the same culture (Figure 3A [i–iv]). Irregular, coral-like multicellular structures and spheroidal multicellular structures are seen upon cytocentrifugation and Giemsa staining (Figure 3A [v]). To maintain the 3D nature of the structures, we fixed them in 4% paraformaldehyde (PFA) and solidified them in Histogel™ to facilitate embedding. Paraffin-embedded sections were stained with H&E. The data in Figure 3A [vi] also show the various sizes and groupings of the multicellular structures. These have a particular tendency to display more organized structures with a central cavity, which suggests a hollow nature, as well as less organized structures. The different cellular arrangements within the multicellular structures were further reflected in the differential pattern of immunostaining of E-cadherin (Figure 3A [vii]) in non-adherent structures. The cell–cell adhesion protein was expressed in a honeycomb-like arrangement in spheroidal multicellular structures, suggesting robust cell–cell adhesion. In contrast, E-cadherin had a grape-like arrangement in irregular multicellular structures, denoting less vigorous cell–cell adhesions. Non-adherent structures, obtained upon serial sectioning of Histogel^TM^/formalin-fixed paraffin embedded tissues (FFPE), confirm the expression of E-Cadherin (Figure 3B [i]) and ovarian cancer biomarker CA125 (Figure 3B [ii]). Finally, Figure 3B [iii] shows heterogeneous expression of p53 in both grape-like and spheroidal multicellular structures. 

### 2.6. Non-Adherent Ovarian Cancer Multicellular Structures of Spheroidal Nature Have the Capacity to Perpetuate the Entire Phenotype when Moved to a New Culture Environment

To determine whether a floating spheroid could recreate the entire phenotype of the culture when placed in a new plating surface, we followed the behavior of a large spheroid observed at the time when it only adhered to the plate upon transference (Figure 3C [i]) and that contained a very small, nascent structure. With time in culture, the multicellular structure developed foci that grew upright aggregates of wide-ranging morphologies. We also observed that the small nascent spheroid became larger. Finally, the original structure gave rise to an adherent component (Figure 3C [ii–iv]). 

### 2.7. PEO1, PEO4, and PEO6 Cells Have Differential Tumorigenicity in Vivo 

We next tested the tumorigenic ability of the following: PEO1, which does not form either foci or non-adherent multicellular structures in culture; PEO4, which forms scattered foci and few non-adherent multicellular structures; and PEO6, which forms both abundant foci and non-adherent multicellular structures. We injected nude mice with a low load of cells (2 × 10^6^ cells) or a high load of cells (20 × 10^6^ cells) (Table 1). PEO1-injected animals did not develop either macroscopic or microscopic disease after 14 months of follow up at any load level. However, PEO4 cells, though they did not display apparent disease at a low load, generated peritoneal carcinomatosis 6 months following intraperitoneal dissemination when injected at a high load. Finally, according to the nature of the progression of the disease, PEO6 developed disease when using a low load of cells after 3.5 months of injection. Overall, we documented that PEO4 and PEO6 diseases were generated around particular areas within the abdominal cavity (Table 2). We concluded that, despite having originated from the same patient, the capacity of these cells to form disease in vivo is of PEO6 > PEO4 > PEO1, which is directly related to their capacity to form foci and multicellular structures in vitro (Figure 1A).

### 2.8. Histopathological Assessment of Intraperitoneal Tumors Derived from PEO4 or PEO6 Cells

#### 2.8.1. Diaphragm 

**Figure 4:**
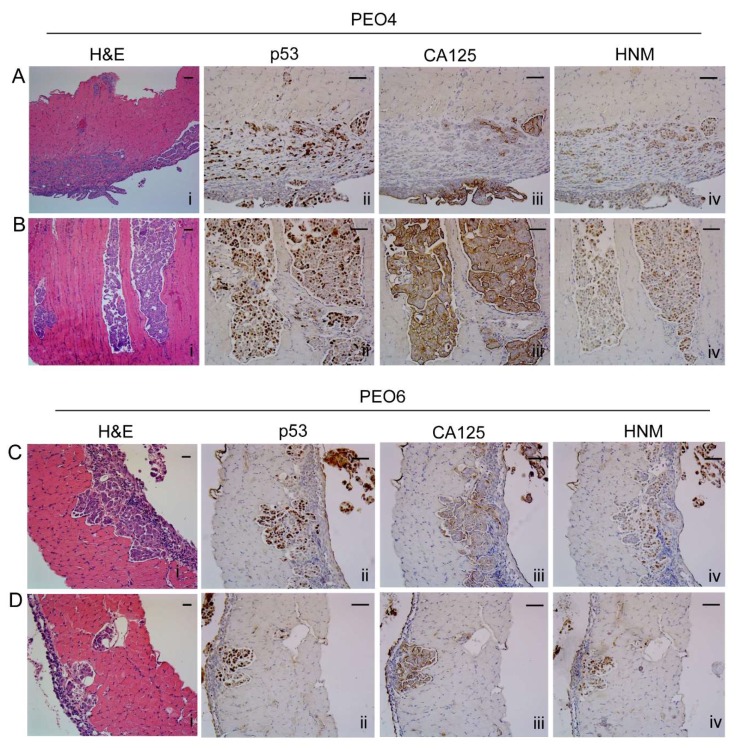
Metastases within the diaphragm. (**A**) Solid pattern of invasion of PEO4 cells visualized by H&E, and expression of p53, CA125, and human nucleoli marker (HNM). (**B**) Micropapillary pattern of invasion. (**C**,**D**) Different morphological features of metastases by PEO6 cells showing mostly an invasive front with slit-like areas that stain positive for p53, CA125, and HNM. Notice the strongest positivity of the markers in the multicellular structures located around the surface of the diaphragm. Scale bars = 50 μm.

The histopathology of the disease reaching the diaphragm was slightly different for PEO4 cells when compared to that of PEO6 cells. PEO4 cells show a compact, solid, expansible pattern that stains positive for mutant p53 and human nucleoli marker (HNM), with differential expression of CA125 that is highlighted mostly at the periphery of the diaphragm (Figure 4A [i–iv]). In addition, Figure 4B [i–iv] shows an infiltrating micropapillary pattern as stained by H&E, mutant p53, CA125, and HNM. For PEO6 cells, images in panels of Figure 4C [i–iv] and Figure 4D [i–iv] show an infiltrating pattern of invasion, which also denote the presence of multicellular structures around the tissue and without apparent implantation. The disease within the diaphragm is limited to microscopic metastases, since there is no apparent gross alteration of the tissue when observed under the stereoscope (Figure S4A–C). However, the microscopic invasion of the parenchyma of the diaphragm was evident for PEO4-injected animals (Figure S4E,H) and for PEO6-injected mice (Figure S4F,I), but not for control animals (Figure S4D,G).

#### 2.8.2. Omentum 

**Figure 5:**
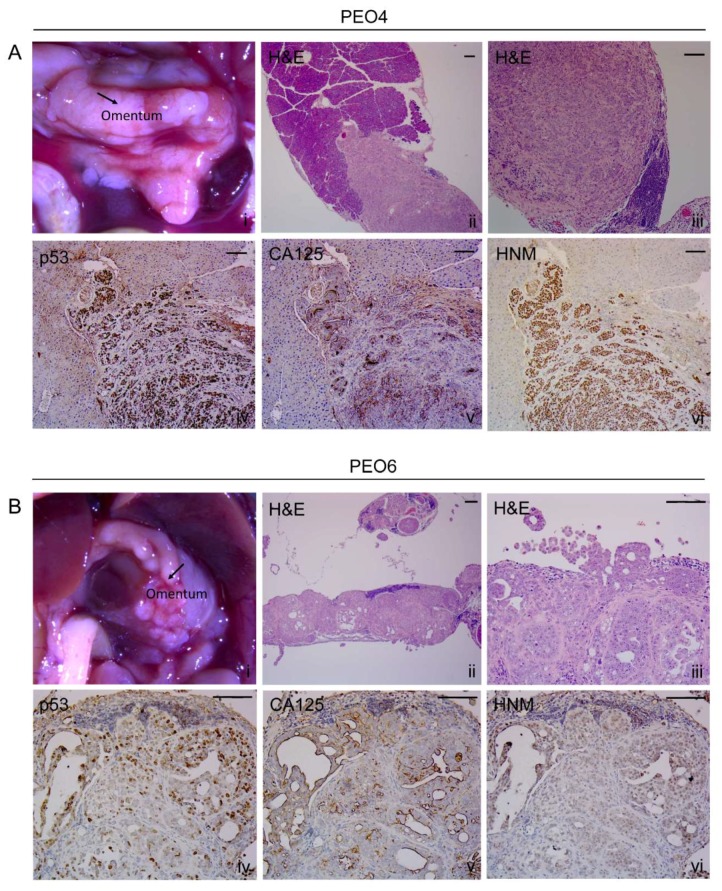
Metastases in the omental area. (**A**) PEO4 cells occupy the omentum and invade in the direction of the pancreas. Images show the positivity of the invasive metastases for p53, CA125, and HNM. Notice that the pattern of expression of cancer cells is very tight or solid. (**B**) Arrangement of PEO6 cells in the omental area depicts a micropapillary phenotype with multicellular structures accumulated toward the periphery of the tissue. Notice that the staining for p53 and HNM is more homogeneous than that of CA125, which is mostly expressed toward open tissue with pseudoglandular areas. Scale bars = 100 μm.

This tissue (Figure 5A [i] and Figure 5B [i]) was heavily invaded and replaced by cancer cells as observed in Figure 5A [ii, iii] for PEO4 and Figure 5B [ii, iii] for PEO6. The arrangement of PEO4 cells within the omentum was characterized by densely packed cells arranged in a solid pattern and highlighted by the expression of mutant p53 (Figure 5A [iv]), CA125 (Figure 5A [v]), and HNM (Figure 5A [vi]). Interestingly, the invasion of PEO4 cells towards the pancreatic area was highlighted by the limited expression of CA125 to very reduced areas where slit-like spaces are scarcely formed (Figure 5A [v]). The PEO6 omental metastases (Figure 5B [i]) are histologically characterized by a micropapillary architecture with some solid areas (Figure 5B [ii,iii]). The staining for p53 (Figure 5B [iv]) and HNM (Figure 5B [vi]) denotes the human nature of the metastatic growth. Expressed CA125 mostly associated with glandular structures in open areas of the tissue not frequently invaded by mouse stroma (Figure 5B [v]).

#### 2.8.3. Ovary 

**Figure 6:**
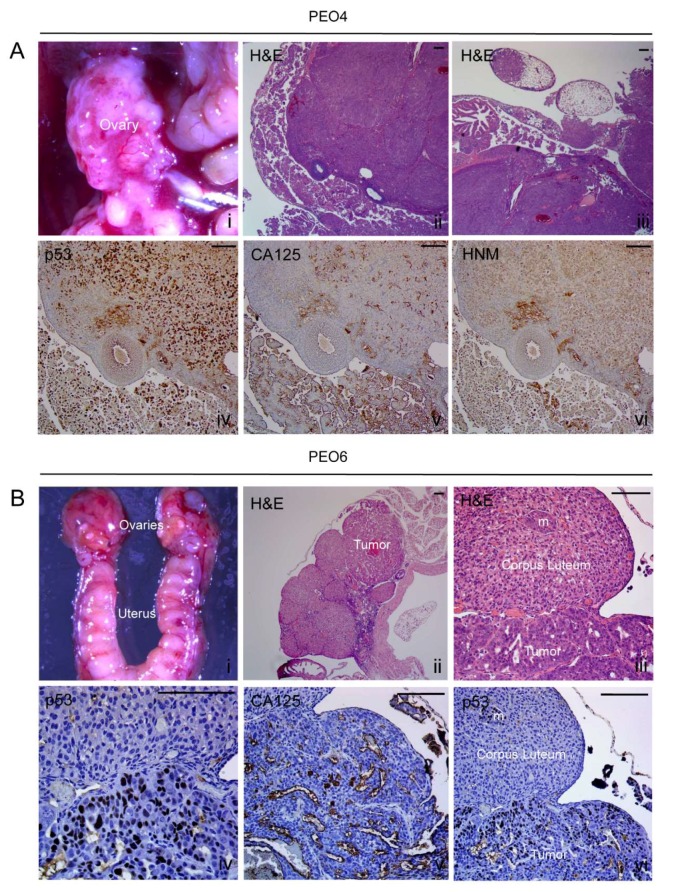
Metastases to the ovaries. (**A**) PEO4 cells occupy the ovary and shows positivity for the expression of mutant p53, CA125, and HNM. (**B**) PEO6 cells formed a tumor within the ovary. Higher magnification shows the clear limit between the luteal tissue and the tumoral area, and the positivity of the tumor cells for p53 and CA125 ([iv,v]). Notice the p53 positive cells identifying an isolated metastasis (m) within the corpus luteum ([vi]). Scale bars = 100 μm.

A high load of PEO4 cells resulted in the formation of a tumor in the ovary (Figure 6A [i]). H&E-stained sections show the structure of the ovary highly compromised by tumor cells (Figure 6A [ii,iii]). Within the solid ovarian structure, p53 and HNM are highly expressed (Figure 6A [iv,vi]. CA125 was largely expressed in multicellular structures found within the bursa that surrounds the ovary but appeared scattered in some areas of tumor cells overlaying the organ (Figure 6A [v]). As for PEO6, they compromised the ovaries with a low load of cells (Figure 6B [i]): there is a clear tumor growth within the ovary (Figure 6B [ii]). At higher magnification, a microscopic growth within a corpus luteum can be observed (Figure 6B [iii]). This growth also stains positive for p53 (Figure 6B [vi]). Solid sheets of tumor cells also stained positive for p53 (Figure 6B [iv]) and express CA125 in slit-like areas only (Figure 6B [v]). For both PEO4- and PEO6-injected animals there was an accumulation of cancerous cells in the fat area that surrounds the ovaries, as demonstrated by their positive staining for p53, CA125, and HNM (Figure S5). Within this tissue, the CA125 patterns of expression clearly identify the homogeneous arrangement of cancer cells of PEO6 origin, when compared to the double pattern observed in PEO4 cells (less differentiated solid areas with light CA125 and cells heavily labeled arranged in more differentiated manner outside the fatty tissue).

#### 2.8.4. Liver 

**Figure 7:**
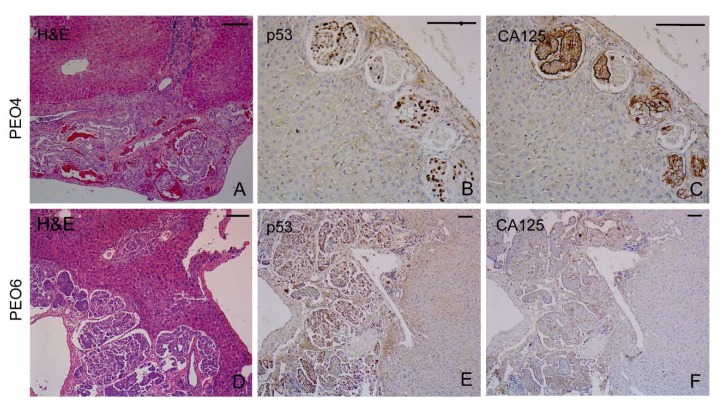
Metastases at the base of the liver. Images show sections of tumoral tissue derived from PEO4 cells (**A**–**C**) or PEO6 cells (**D**–**F**). PEO4 cells show positivity for p53 and CA125 (**B**,**C**), whereas similar results are observed for PEO6 cells (**E**,**F**). Scale bars = 50 μm.

The capsule of the liver was often infiltrated by PEO4 cells or PEO6 cells gaining access to the parenchyma. These cells exhibit the characteristic slit-like arrangement (Figure 7A,D). The papillary areas were also positive for p53 (Figure 7B,E) and were decorated with CA125 (Figure 7C,F).

#### 2.8.5. Peritoneal Wall

A tumor of PEO6 cells is clearly observed invading the wall of the peritoneum (Figure S6A). The accumulation of cellular aggregates by the wall of the peritoneum depicts a micropapillary pattern (Figure S6B,C) that stains positive for p53 (Figure S6D), CA125 (Figure S6E), and HNM (Figure S6F). The arrangement of multicellular structures in this region resembles the arrangement cells have spontaneously in vitro (Figure 1, Figure 2 and Figure 3).

### 2.9. Orthometastatic Injection of PEO6 Multicellular Structures Is Sufficient to Trigger the Development of Bloody Ascites and Peritoneal Disease 

**Figure 8:**
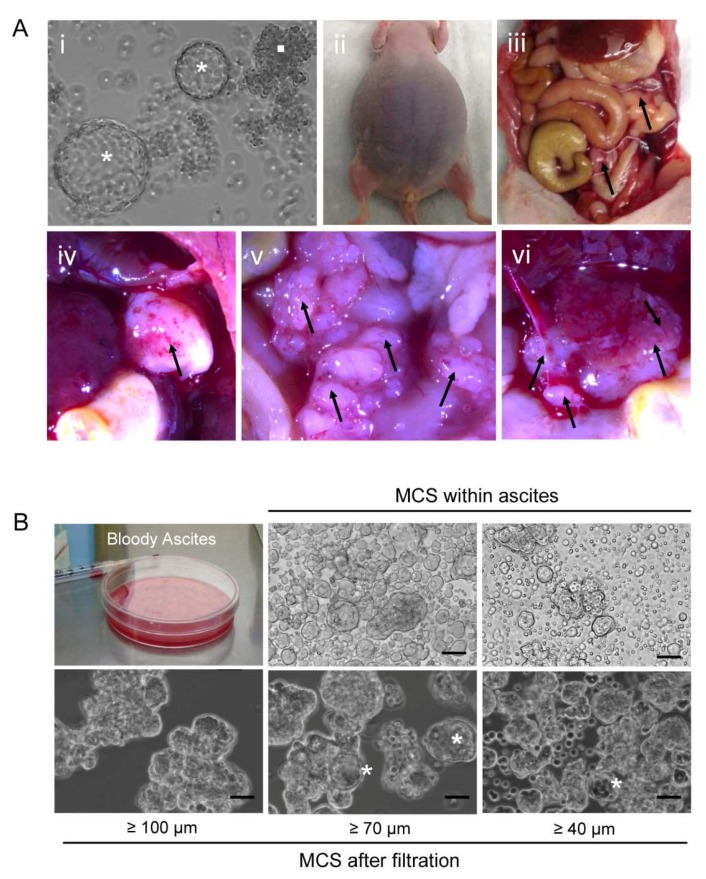
(**A**) Representative images depicting tumor burden in a nude mouse sacrificed after being injected in the peritoneal cavity with a mix of irregular (*square* in [i]) and spheroidal (*asterisks* in [i]) multicellular structures. The image in [i] was obtained using an inverted microscope. Images in [ii] and [iii] were taken with a digital camera. Images in [iv–vi] were obtained using a high-power stereoscopic microscope. Areas with arrows denote the presence of tumors, as an isolated entity [iv], in the omental area [v], and in the base of the liver [vi]. (**B**) Multicellular structures growing in the peritoneal cavity of nude mice are bloody and, upon filtration of the blood cells, show different sizes; scale bars = 25 μm.

**Figure 9:**
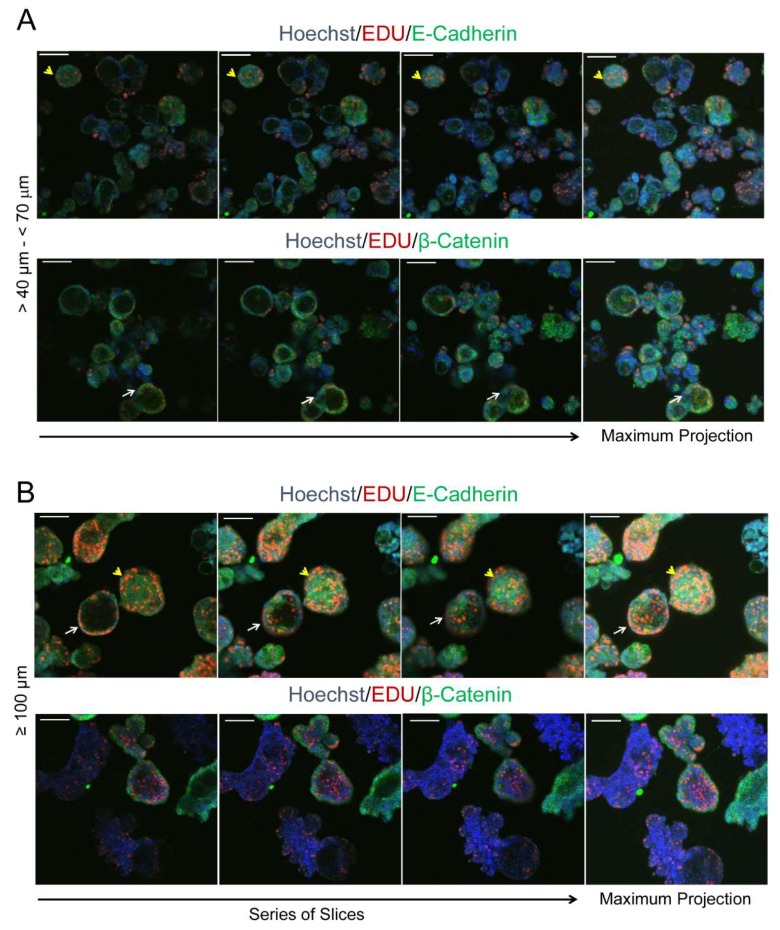
Multicellular structures isolated from peritoneal effusions as evaluated by inverted confocal microscopy. In (**A**,**B**) yellow *arrowheads* denote compact multicellular structures; white *arrows* show hollow multicellular structures. EdU labelling in red denotes cells synthesizing DNA. Green staining denotes expression of E-cadherin and β-catenin. Blue, Hoechst staining. Scale bars = 100 μm.

We next studied whether the self-assembled multicellular structures in culture were sufficient to develop disease in immunosuppressed mice when placed within the peritoneal cavity in direct contact with the organs to which the illness normally metastasizes (i.e., orthometastatic placement). We injected, into the lower pelvic cavity of three nude mice, a mixture of ~10,000 irregular and spheroidal multicellular structures spontaneously formed from cultured PEO6 cells (Figure 8A [i]). The animals respectively met the criteria for euthanasia 4, 6, and 7 months later, thus averaging 5.67 months following cellular injection: this was due to the large distension of the abdomen (Figure 8A [ii]), breathing difficulties, and reduced mobility, all consequences of the accumulation of abundant bloody ascites (Figure 8A [ii] and Figure 8B). The solid growths in the abdominal cavity were discrete and mainly found in the omental region (Figure 8A [iii–v]). Small tumor foci were also found in the liver (Figure 8A [vi]). One of the three animals also developed pleural effusions. The accumulated ascites, after filtration from blood cells, fibroblasts, and mesenchymal cells, revealed the presence of floating multicellular structures of diverse sizes ranging from approximately 40 μm to over 100 μm (Figure 8B). We then used an inverted fluorescence confocal microscope to study the proliferative capacity of the isolated multicellular structures via EdU incorporation, the nature of the cell–cell adhesions via labeling for E-cadherin, and for its associated molecule β-catenin. We also assessed the hollow or compact nature of the multicellular structures (Figure 9A,B). We found high heterogeneity in the multicellular structures isolated from the peritoneal effusions in terms of size, proliferative capacity, and cell–cell adhesion molecules. We also demonstrated, using serial confocal slices, that regardless of size and structure, either irregular or spheroidal, all multicellular structures have regions of staining for EdU incorporation. We also observed that the tendency to form hollow structures is more predominant in spheroidal than in irregular multicellular structures; nevertheless, we observed irregular structures and spheroidal structures that are compact as well. Finally, we observed that the expression of β-catenin is lost in some of the larger irregular multicellular structures. 

**Figure 10:**
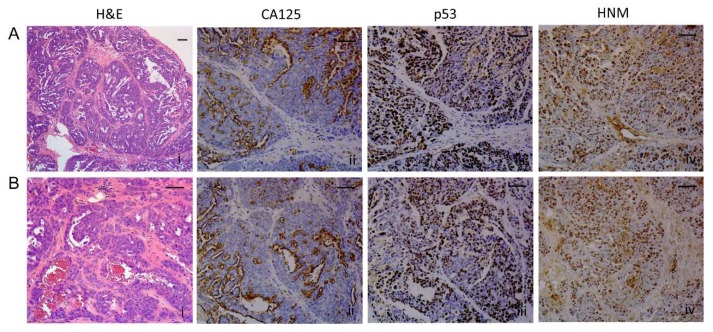
Histopathological analysis of the nodular growth shown in Figure 8A [iv]. (**A**) and (**B**) Different images showing tumor cells arranged in a pseudoglandular architecture with slit-like areas surrounded by fibrovascular stroma and having a homogeneous expression of p53 and HNM. The expression of CA125, however, is more heterogeneous and limited to the open slit-like areas. Scale bars = 50 μm.

Next, we studied the histopathology of the peritoneal mass formed in a nude mouse upon injection of PEO6 multicellular structures shown in Figure 8A [iv]. Figure 10A [i] and Figure 10B [i] display the microscopic aspect of the tumor that shows a pseudoglandular architecture with irregular slit-like spaces subdivided by some mouse fibrovascular stroma. The mass retained the expression of CA125 (Figure 10A [ii] and Figure 10B [ii]) and of p53 (Figure 10A [iii] and Figure 10B [iii]). Whereas the latter is mostly homogenously expressed throughout the tumoral tissue, the former is expressed mainly in tumor cells bordering the slit-like spaces. Staining for HNM in Figure 10A [iv] and Figure 10B [iv] confirms the human nature of the tumoral cells embedded within the mouse stromal tissue. The glandular aspect with slit-like areas (Figure S7B,C), characteristic of HGSOC, was also observed clearly in nodules formed within the omental region (Figure S7A). The metastatic growth shows the typical phenotype of cells completely taking over the omentum. The cells stain for HNM (Figure S7F), mutant p53 (Figure S7E), and biomarker CA125 (Figure S7D), which is expressed towards the lumen of the slit-like areas. Finally, we observed the capacity of multicellular structures to reach outside the abdominal cavity by colonizing the parenchyma of the lung, where the pseudoglandular nature of the tumor is associated with the expression of mutant p53, CA125, and HNM observed at low magnification (Figure S8, panels A–D) or higher power (Figure S8, panels E–H). 

## 3. Discussion

The multicellular aggregates in peritoneal effusions (ascites) and pleural effusions of patients with ovarian cancer were characterized in 1987 by Allen and colleagues [23]. These aggregates were depicted as highly heterogeneous between patients and even within the effusions of a single patient. In particular, for HGSOC, the clustered cells were described as either compact or loosely adherent with different degrees of budding and an often-visible central lumen. More importantly, it was shown that the cell–cell interaction was actively mediated by desmosomes which provide strong inter-cellular adhesion and bonding with the intermediate filament cytoskeleton; this confers mechanical strength upon the structures. These early observations are in line with the multicellular structures we observed spontaneously formed under adherent culture conditions, and within the ascites of immunosuppressed mice upon injection of HGSOC cells. 

The spontaneous formation of spheroids budding from monolayers was observed in A2780, SKOV-3, and HEY cells [24], which do not represent the most frequent cases of ovarian cancer—HGSOC [25]. Those authors showed that the cells, cultured in standard media under adhesion-prone conditions, develop upward buds from the monolayer, which finally detach from the culture surface and lead to the formation of compact multicellular structures. The heterogeneity of the multicellular structures, derived particularly from high-grade serous PEO6 cells, was depicted in the size of the structures ranging from less than 40 μm to more than 100 μm; some structures were less organized or grape-like, whereas others were more spheroidal; some of them showed a hollow center, while others were compact as demonstrated using inverted confocal microscopy. The heterogeneity was also depicted by the sites within the structures where cells were multiplying, dying, or expressing the epithelial E-cadherin-β-catenin complex. The expression of E-cadherin and β-catenin highly suggests that multicellular aggregates have an active molecular program to create the adhesive environment for keeping cells together. Our data support the observation that E-cadherin was shown up-regulated in ovarian cancer serous cellular effusions when compared to primary ovarian cancer tumors, likely providing survival advantage [26]. In terms of the mechanisms of aggregate implantation, there are studies in which ovarian cancer multicellular structures force themselves through the mesothelial cell layer of the peritoneum lining abdominal organs. They do so without undergoing previous disaggregation, yet expressing more mesenchymal markers than those that are not competent for clearance, which, conversely, express more epithelial markers [27,28]. However, there is also a possibility that cells arranged as highly epithelial spheroids metastasize via a slightly different mechanism. We demonstrated that PEO6 spheroids have metastatic capacity in vivo despite the fact that previous authors considered PEO6 as clearance incompetent [27,28]. Thus, it is possible that, in addition to multicellular structures passing through the mesothelium, some structures have a different modality of adhesion and invasion involving disaggregation of the clusters followed by adhesion and invasion. Supporting this idea, it was shown ex vivo that multicellular structures from patient-derived ascites can adhere to and disaggregate on live human mesothelial cell monolayers, occasionally invading them [29,30]. In summary, it seems that within the ascites there is a dynamic niche composed of a heterogeneous population of multicellular structures, some with the capacity to invade organs, and others with the capacity to act as reservoirs or niches of the disease in the non-adherent environment of the ascites as we proposed earlier [31]. 

We found that the capacity to form foci that detach leading to the formation of live, floating multicellular structures increases when we used cells taken from the same patient when the disease was more advanced. Thus, the capacity to form foci was higher for PEO6 cells, taken from ascites of the patient before death, than for PEO4 cells, derived from ascites of the patient when platinum resistant. The least prone to form budding foci were the PEO1 cells derived from the ascites of the patient when she was still platinum-sensitive. A similar trend was depicted by PEO23 cells obtained from the ascites of a recurrent patient, which were more capable to form multicellular structures than PEO14 cells that were derived from the same patient when the disease was first diagnosed and before receiving chemotherapy. 

One aspect that favors the higher tumorigenicity of floating, live cells in ascites and in secondary metastasis is their higher percentage of stemness markers when compared to the primary tumors [32,33,34]; furthermore, cells isolated based on stem cell markers are capable of forming compact spheroids in vitro if adherence is prevented [32], indicating that cells with cell renewal capacity may find a niche within aggregates or spheroids making them a vehicle of malignancy. Stem cell markers are also enriched in ovarian cancer cells because of short-term cisplatin/paclitaxel chemotherapeutic selection [20] or multiple passaging in culture of non-adherent cellular aggregates [35]. These results are in agreement with our data showing higher capacity to form spontaneous multicellular aggregates—even under adherent-prone conditions—of cells isolated from patients after development of platinum-resistance (e.g., PEO6 and PEO23 cells). 

We observed that cancer stem cell markers, CD133 and CD44, are expressed in all cell types studied, though at different levels. When progressing along the disease, CD44 was less abundant, whereas CD133 increased its expression. An overlap between the expression of these biomarkers was reported for prostate cancer [36], whereas an independent expression was reported for breast cancer [37]. Our data correlate with data from colon cancer cell lines derived from the same patient, in which the early stage cells were almost all CD44 positive, while only a very small percentage were CD133 positive. In the later stage of the disease, however, CD133-positive cells were more abundant than CD44-expressing cells [38]. The overlap or independence of CD133 and CD44 in HGSOC is unclear [39]. It has been shown, in different cell populations within the ascites of advanced ovarian serous adenocarcinomas, that the expression of stem cell surface markers does not necessarily coincide with the expression of transcription factors driving stemness [19,40]. Further studies need to be done with PEO1, PEO4, and PEO6 cell lines to determine, between CD44 and CD133, which is a surface marker and which one provides stemness, or whether both have mixed properties in ovarian cancer stem cells along disease progression [32,39,41,42,43]. Moreover, whether the selective pressure that chemotherapy had on the tumor cells along disease evolution is related to the distinctive pattern of expression of CD133 and CD44, deserves to be studied. 

We show that serous ovarian cancer cells can form multicellular structures that float in culture dishes despite being offered optimal conditions to adhere to the plate. However, this capacity was poorer for PEO1 cells when compared with PEO4 cells or PEO6 cells. This is interesting, since all cell lines were originally established from cellular ascites of the same patient with noticeable symptomatology. It is likely that the selective pressure of the chemotherapeutic schedule the patient was receiving before each cell line was established played a role in the development of different cellular behaviors that became reflected in our in vitro and in vivo results. 

To develop multicellular structures in vitro, investigators have used support systems that create conditions in which the adhesive forces between cells are greater than for the substrate on which they are plated. Exceptions include the colon carcinoma cell line LIM1863, which shows spontaneous capacity to form spheroidal structures in culture (reviewed in [44]), and the previously described non-serous ovarian cancer lines A2780, SKOV-3, and HEY [24]. Such support systems include the use of non- or low-adherence plates, or gravity and collision forces to promote cell–cell adhesion, such as the hanging-drop method or the rotary cell culture system (reviewed in [45]). These models, however, assume that mono-dispersed ovarian cancer cells, when gathered together either by enforced gravity or prevention of adhesion, mimic the program of assembly followed by ovarian cancer multicellular structures found within malignant effusions (reviewed in [46]). Studying the multicellular structures found in ascites should help unveil the mechanistic process of spheroid formation without the caveats of the methods that force cellular aggregation. 

We prove that the formation of multicellular structures is a complex program, leading, for instance, to the coexistence of multicellular irregular aggregates and more organized spheroids within the same cellular entity. These results highlight the heterogeneity, adaptability, and plasticity of the cells causing the disease. When we orthometastatically re-propagated the irregular and organized multicellular structures in nude mice, we observed that the structures were sufficient to trigger the formation, in vivo, of both types of entities free floating in the peritoneal cavity, having assorted sizes, replicative capacity, and heterogeneity in terms of their either compact/solid or hollow anatomy. The concept that a major driver of ovarian cancer progression might reside within multicellular structures found in suspension has been gaining presence in the literature in the past 20 years [19,26,29,30,46,47,48,49,50,51]. Our results support the concept that multicellular structures found within the liquid facet of the disease represent active products of disease selection and critical drivers of disease pathogenesis. The abundant evidence currently present in the literature makes clear that similar relevance should be given to solid and floating entities. They are part of a dynamic system that communicates to perpetuate the disease. Altering the communication system likely taking place in a paracrine fashion between adherent and floating cells may be the key target of new treatment approaches to interrupt disease progression. 

Our results were obtained longitudinally in cell lines generated from the same individual along disease progression; however, due to the heterogeneity of the disease, we cannot ascertain whether HGSOC cells serially isolated from ascites of other patients will behave as PEO1, PEO4 and PEO6 cells do. After all, these three cell lines represent a single patient.

The histopathological aspects of ovarian cancer recreated in immunosuppressed mice within the abdominal cavity (i.e., orthotopically)—the natural environment where ovarian cancer progresses—has been described. First, in 1984, it was shown that OVCAR-3 cells were found to be invasive in nude mice forming i.p. tumors with ascites and pulmonary metastasis [52]. Cells in the ascites showed acinar and papillary-like structures when found as groups and sheets of adenocarcinoma cells. Solid tumors showed glandular and papillary aspect with numerous mitotic figures. Later on, in 1987, i.p. tumors were generated in nude mice using JAM, TRI70, SKOV-3, or OAW42 cell lines [53]. In a comprehensive study by Shaw and colleagues [54] in 2004, nude mice injected i.p. with a high load of eight different cell lines (A2780-s, A2780-cp, ES-2, HEY, OCC-1, OVCA429, SKOV-3, and OV2008) developed disease. The animals consequently experienced median survival, ranging from 16 to 105 days. Most of the previously described cell lines, however, are not at the top of the list of what has, since the publication of the TCGA data on HGSOC [55], genetically been considered as belonging to this histotype [17,25]. Since the TCGA, however, when i.p. tumors have been developed in nude mice with cells genetically resembling HGSOC, the results have been controversial. The cells that ranked higher in their genomic resemblance to HGSOC, such as Kuramochi and OVSAHO, did not grow well [14,15]. Herein we provide evidence that the nude mouse represents a suitable model for i.p. disease development when using certain types of high-grade serous cells, such as the case of PEO4 and PEO6 cells representing advanced disease [13,56]. Furthermore, whereas peritoneal growth caused by non-serous ovarian cancer cells seem to be represented by large masses [31], those we observe with high-grade serous lines show discrete solid growths but with clearly established microscopic metastases. We provide evidence that the histotype generated by these cell lines in the peritoneal cavity of nude mice faithfully represents the micropapillary nature observed in HGSOC patients in target organs such as the omentum, diaphragm, ovaries and peritoneal wall, with the accompanying abundant bloody ascites. 

The time it took PEO4 and PEO6 cells to develop intraperitoneal disease, regardless of the cell load (3.5–6 months), could be considered a long time, since the majority of studies use a 3-month timeline to end experiments [14,15]. However, the take time of PEO4/6 tumors is not different from the time it takes fresh material (ascites or slurry of solid tumors) obtained from patients to develop tumors in immunosuppressed mice. For instance, tumor-derived xenografts generated from a patient’s ascites developed disease in highly immunosuppressed mice after 2–12 months following i.p. transplantation [16]; in another case, 0.3–0.5 cm^3^ of tumor slurry caused disease in NSG mice after 4–6 months following i.p. transplantation [57]; furthermore, a slur of a patient’s tumor containing tumor cells, fibroblasts, and T cells were engrafted i.p. into NSG mice ranging from 2.6 to 4.6 months [58]. Finally, a fresh tumor from the ascites of a patient intraperitoneally injected into female NOD/SCID mice was reported to develop abdominal distention within 6 months after inoculation of a high load of cells (1 × 10^7^) [59]. Thus, it seems reasonable that it takes time for the human cells to stimulate the mouse stromal cells to support new growths. 

The development of intra-abdominal disease upon injection of multicellular ovarian cancer structures has been less studied. In 2007, Zietarska and colleagues, using OV90 ovarian cancer spheroids induced by cell aggregation via the hanging drop method, reported the development of intraperitoneal tumors in SCID mice in the totality of the animals after approximately 2 months of injection [50]. In our study, we demonstrated the tumorigenic capacity of spontaneously formed multicellular structures that do not undergo forced aggregation and that are derived from ovarian cancer cells isolated from the same patient along disease evolution. We provide detailed anatomical locations and histopathological description of the tumors formed, as well as demonstrate that nude mice, which are immunosuppressed only in terms of T-cell function, can be used as a reliable model of disease progression driven by non-adherent multicellular structures, if sufficient time is given for the disease to develop. 

Notably, we found a difference in the load of cells needed to develop peritoneal disease, with a low load for PEO6 cells, a high load for PEO4 cells, while no disease was achieved even with a high load of PEO1 cells. We cannot rule out, however, that such a high load of PEO1 cells would not develop intra-abdominal tumors in mice having heavier immunosuppression than our nude mice, as a recent work shows positive formation of subcutaneous tumors in NSG mice injected with GFP-labeled PEO1 cells [60]. 

It is possible that PEO6 cells, which were obtained from a heavily treated patient, may undergo a further selection from PEO4 cells that were obtained from the patient before the last round of chemotherapy, while PEO1 cells may have not been selected sufficiently by chemotherapy to be enriched of tumorigenic clones. In line with this reasoning, the non-adherent component derived from ascites of chemoresistant patients was shown to have an enriched epithelial phenotype (high E-cadherin, high EpCAM, and high CA125) with tumorigenic capacity, when compared to the non-tumorigenic capacity of the adherent phenotype derived from the same ascites, which encompasses a more mesenchymal phenotype [19].

When we studied the expression of antigen CA125, we found that its pattern of staining was highly heterogeneous. In PEO1/4/6 cells maintained in culture, CA125 was expressed in all cell types, yet with a major abundance aligned with disease evolution. In vivo, within the tumors, the expression of CA125 was mostly limited to the apical region of cells facing slit-like spaces generated in between solid sheets of tumor cells surrounded by fibrovascular stroma. When tumor cells were located deep within homogeneous solid patterns devoid of slit-like fenestrations, they usually did not express CA125. In contrast, when cells were found gathered as multicellular structures free-floating in open spaces surrounding target organs—e.g., within the bursa around the ovaries—CA125 was highly expressed. This coincides with the high expression of CA125 observed in vitro in cells spontaneously forming multicellular aggregates in suspension. It is likely that HGSOC cells express CA125 to face or invade open spaces, generating the adequate environment for neo-angiogenesis. This selected pattern of expression may also be related to the oncogenic functions of CA125 (a.k.a., MUC16, a glycoprotein), such as protecting cells from immunological attacks or promoting metastases by binding to mesothelin produced by mesothelial cells that line the peritoneal cavity (reviewed in [61]).

## 4. Materials and Methods 

### 4.1. Cell Culture

The PEO1, PEO4, and PEO6 cell lines were established sequentially from the same patient and reported first in 1988 [12]. PEO1 were derived from the ascites of a patient diagnosed with a poorly differentiated serous adenocarcinoma. The cells were collected after initial treatment with cisplatin, 5-fluorouracil, and chlorambucil. PEO4 cells were isolated from ascites of the same patient after she developed resistance to the previous chemotherapeutics, whereas PEO6 cells were isolated from ascites collected prior to her death. We originally obtained the cells from Dr. Taniguchi (Fred Hutchinson Cancer Center, University of Washington, Seattle, WA, USA) with the written consent of the originator, Dr. Langdon (Edinburgh Cancer Research Centre, Edinburgh, UK) [62]. Longitudinally paired cell lines PEO14 and PEO23, described by Langdon and colleagues in 1988 [12], were obtained from Culture Collections, Public Health England (Porton Down, Salisbury, UK). The genetic analysis indicating fidelity of high-grade serous ovarian carcinoma (HGSOC) for these cell lines was reported over twenty years later [13]. The cells, despite originating from the same patients, were shown to have karyotype divergence. Their genetic diversity was also characterized by high-resolution array comparative genome hybridization (CGH). The cells show multiple copy number alterations consistent with the origin of HGSOC. Because PEO1, PEO4, PEO6, PEO14, and PEO23 cells were all reported to have high genetic fidelity to HGSOC [13,25], we standardized their culture conditions using RPMI-1640 (Mediatech, Hendon, VA, USA) supplemented with 10% fetal bovine serum (Atlanta Biologicals, Lawrenceville, GA, USA), 10 mM HEPES (Mediatech), 4 mM L-glutamine (Mediatech), 1 mM sodium pyruvate (Mediatech), 100 IU penicillin (Mediatech), 100 μg/mL streptomycin (Mediatech), and 0.01 mg/mL human insulin (Roche, Indianapolis, IN, USA). Cultures were done in plates with standard adherence properties at 37 °C and under a humidified atmosphere of 95% air/5% CO_2_. 

### 4.2. Cell Line Authentication

All cell lines were authenticated using autosomal short tandem repeat [STR] profiling markers showing a ≥80% match between the cells used in this study and the original cell lines with profiles suitable for verification in references databases. The authentication was done in the cell line authentication core facility of the University of Arizona (Tucson, AZ, USA) (https://uagc.arl.arizona.edu/faq/cell-line-authentication). The cell line authentication report is available in Appendix A. 

### 4.3. Phase Contrast Microscopy

Cells were cultured for 10 days under adherent conditions in standard tissue culture plates. During the culture period, the cells, originally plated monodispersed, adhered to the plate forming a monolayer, yet they also formed tri-dimensional (3D) foci. Phase contrast images of developed spheres were obtained using a Zeiss Axiovert 200 M inverted microscope with an AxioCam HRm camera (Carl Zeiss Meditec AG, Jena, Germany). 

### 4.4. Cytospin Preparations

Floating multicellular structures were studied after being cytocentrifuged (Cytofuge 2, StatSpin Inc., Norwood, MD, USA). Thereafter, the structures were fixed in 4% PFA and stained with Giemsa. In other studies, the multicellular structures were fixed in 4% PFA and solidified with Histogel^TM^ (Richard-Allan Scientific, Kalamazoo, MI, USA) before paraffin embedding, and followed by 5 μm sectioning before staining with H&E. 

### 4.5. Fluorescence Microscopy

For a live/dead^®^ viability/cytotoxicity assay, we followed a protocol we previously described in detail [63]: multicellular structures were incubated for 45 min at room temperature (RT) and without fixation in the presence of 2 μM calcein AM (Molecular Probes, Eugene, OR, USA) and 4 μM ethidium homodimer 1 (EthD-1) (Molecular Probes). Calcein AM fluoresces green when cells are alive due to their esterase activity, while EthD-1 only enters dying cells and stains the DNA that fluoresces in red. Images were obtained with a confocal Olympus FV1000 microscope with FluoView^®^ software (Olympus Corporation, Tokyo, Japan). 

In some experiments, adherent as well as multicellular structures growing in suspension were subjected to the expression of the epithelial/membrane markers E-cadherin and β-catenin. In such experiments, counterstaining was done using DAPI (Molecular Probes) or Hoechst ( Invitrogen, Carlsbad, CA, USA). 

In experiments in which the morphological nature of the structures was studied (i.e., whether compact or hollow), live cultures containing multicellular structures were filtered using meshes that separate structures ranging from 40–70 μm and those larger than 100 μm. Chamber slides containing multicellular structures in suspension and without fixation were exposed to 10 μM of the Click-iT^®^ EdU Imaging reagent (Invitrogen) for 14 h. EdU (5-ethynyl-2′-deoxyuridine) is a nucleoside analog of thymidine that is incorporated into the DNA during its active synthesis. The detection of EdU is achieved by a small size Alexa Fluor azide that gains access to the nucleotide without the need to denature the DNA or to use antibodies. The structures were fixed in 4% PFA and further incubated with primary antibodies to detect E-cadherin or β-catenin, using Hoechst as counterstaining. To detect E-cadherin or β-catenin, a secondary antibody linked to Alexa Fluor 488 was used (refer to Table S1 for source and dilution of the antibodies). The structures were imaged while floating using a Nikon Eclipse Ti inverted confocal microscope using the 3D Z-series properties (Nikon Instruments Inc., Melville, NY, USA) and an Andor iXon 897 camera. 

### 4.6. Immunocytochemistry and Immunohistochemistry

To study the expression of biomarkers of HGSOC, cells were cultured in chamber slides, washed in PBS, fixed with 4% PFA, and subjected to immunostaining for p53, WT-1, PAX8, CA125, HNF1β, ARID1A, E-cadherin, vimentin, CA125, CD44, and CD133. The list of antibodies, sources, and dilutions are depicted in Table S1. Permeabilization was done with 0.5% Triton-100 for 20 min at RT, followed by washing, and further incubation of the cells with 2.5% normal horse serum for 30 min at RT to reduce the non-specific binding. Cells were incubated with the specific primary antibodies in a moist incubation chamber for either 1 h at RT or overnight at 4 °C. Endogenous peroxidase activity was blocked with 3% H_2_O_2_ for 20 min at RT following incubation with secondary antibodies. 

For immunohistochemistry done in formalin-fixed, paraffin-embedded tissues (FFPE), antigen retrieval to detect p53 was carried out by placing tissue sections in a solution of 10 mM Tris/1 mM EDTA/0.05% Tween-20, pH 9.0, for 30 min in a steamer, followed by cool-down at RT for 20 min. For CA125 and anti-human nucleoli marker (HNM) expression, antigen retrieval was done with 10 mM Tri-sodium citrate/0.05% Tween-20, pH 6. Permeabilization, reduction of non-specific binding, incubation with primary and secondary antibodies, were as previously described for immunocytochemistry. 

Specific peroxidase activity was developed with ImmPACT DAB Peroxidase (#SK-4105) (Vector Laboratories, Burlingame, CA, USA). In negative controls, the primary antibody was replaced with 2.5% normal horse serum (Vector Laboratories) (see examples of negative controls performed for the antigens studied in Figures S9–S16). Counterstaining was achieved using hematoxylin QS (modified Mayer’s formula) (Vector Laboratories). Color images were obtained using an Amscope microscope with Amscope Software 3.7 (United Scope LLC, Irvine, CA, USA). 

### 4.7. Studies in Immunosuppressed Mice

In vivo studies were done following approval by the Institutional Animal Care and Use Committee (study protocols 78-12-10-13C (University of South Dakota) and 2017-7909 (McGill University)). Immunodeficient (athymic nude-Foxn1^nu^) female mice (Harlan) at 6 to 8 weeks of age (~23 g in weight) were inoculated i.p. in the right lower pelvic cavity with either of two different loads of monodispersed cells (2 × 10^6^ (PEO1, PEO4, PEO6) or 20 × 10^6^ (PEO1, PEO4)), or ~10,000 irregular and spheroidal multicellular structures spontaneously formed from cultured cells and containing an equivalent amount to ~2 × 10^6^ monodispersed cells (PEO6). The animals were monitored until reaching euthanasia criteria as per approved protocol (weight loss >15%, weight gain >5 g, presence of abdominal distention that affects mobility, respiratory distress, anorexia, and/or diarrhea [54]). At sacrifice, peritoneal fluid was collected, and gross images of the peritoneal cavity were taken with a Leica M165 FC stereomicroscope (Leica Microsystems, Concord, ON, Canada). Then, tumoral tissues were isolated, fixed in 4% PFA, and embedded in paraffin. Five μm sections were obtained and stained with H&E. The slides were analyzed blindly by two pathologists to confirm the diagnoses of HGSOC, as recreated in the immunosuppressed mice. Some of the same biomarkers of HGSOC, tested in the isolated cells while maintained in culture, were studied in the tumors that developed in the immunosuppressed animals. 

## 5. Conclusions

This paper demonstrates that a highly adherent surface does not impede the development of multicellular structures of poor organization or more organized spheroidal ones, which seem to establish a reversible equilibrium with adherent cells. The paper also contends that the higher the capacity of HGSOC cells to develop free-floating, multicellular structures in culture, the easier it was for cells, once injected into nude mice, to develop solid metastatic growths in different sites of the pelvic cavity and a liquid component composed of bloody ascites with a mixture of irregular and organized multicellular structures. This proof of principle is to be corroborated using cells isolated from ascites of multiple HGSOC patients; however, developing therapeutic interventions to interrupt formation of multicellular structures free-floating in the peritoneal fluid may be an efficient manner of interrupting disease recurrence. Furthermore, this investigation suggests that the in vitro, spontaneous, multicellular-structure-forming capacity of epithelial ovarian cancer cells obtained from ascites of patients diagnosed with HGSOC may be used to predict their aggressiveness and, therefore, more effectively guide prognosis. 

## Figures and Tables

**Table 1:** Incidence of peritoneal disease in animals injected with different loads of HGSOC cells.

Group	Ascites	Peritoneal Disease	Time to Euthanasia (Months)	Type of Ascites
Low load-PEO1 cells	0/3	No	14	N/A
High load-PEO1 cells	0/3	No	14	N/A
Low load-PEO4 cells	0/3	No	14	N/A
High load-PEO4 cells	3/3	Yes	6	Bloody with MCS
Low load-PEO6 cells	3/3	Yes	3.5	Bloody with MCS

Low load = 2 × 10^6^ cells; high load = 20 × 10^6^ cells; MCS = multicellular structures; N/A = non-applicable.

**Table 2:** Most frequent areas colonized upon i.p. administration of HGSOC cells.

Location of Mets	PEO4 Cells	PEO6 Cells
Isolated masses	1/3	3/6
Omental-pancreatic area	3/3	6/6
Base of the liver	2/3	3/6
Diaphragm	3/3	2/6
Ovary-oviduct-uterine area	3/3	5/6
Peritoneal wall	1/3	4/6

i.p.: intraperitoneal; mets: metastases. Numbers represent the number of animals with metastases of the total of animals studied for each cell line.

## Supplementary Materials

The following are available online at https://www.mdpi.com/2072-6694/12/3/699/s1, Figure S1: PEO14 and PEO23 cells depict a flat, adherent component and 3D foci, Figure S2: Multicellular structures express p53 and WT1, and are negative for the clear carcinoma cell marker HNF1β, Figure S3: CD133 positive clusters of non-adherent PEO6 cells, Figure S4: Images denote the presence of small foci of disease in the diaphragm of animals injected with either PEO4 or PEO6 cells, Figure S5: Metastasis taking the fat that surrounds the ovary, Figure S6: PEO6 cells arranged as a tumor next to the peritoneal wall, Figure S7: Macroscopic and microscopic view of omental metastasis generated when nude mice were injected with multicellular structures from PEO6 cells, Figure S8: Lung metastasis, Figures S9–S16: Negative controls for the antigens studied via immunocytochemistry and immunohistochemistry, Table S1: Source and dilutions of antibodies used.
