# Supplementary material for: The Capacity of High-Grade Serous Ovarian Cancer Cells to Form Multicellular Structures Spontaneously along Disease Progression Correlates with Their Orthotopic Tumorigenicity in Immunosuppressed Mice
Source: Cancers (Basel). 2020 Mar 16;12(3):699. doi: 10.3390/cancers12030699 (PMC7140084; doi:10.3390/cancers12030699)
Supplement: Supplementary file 1 [file cancers-12-00699-s001.pdf]

Supplementary Material

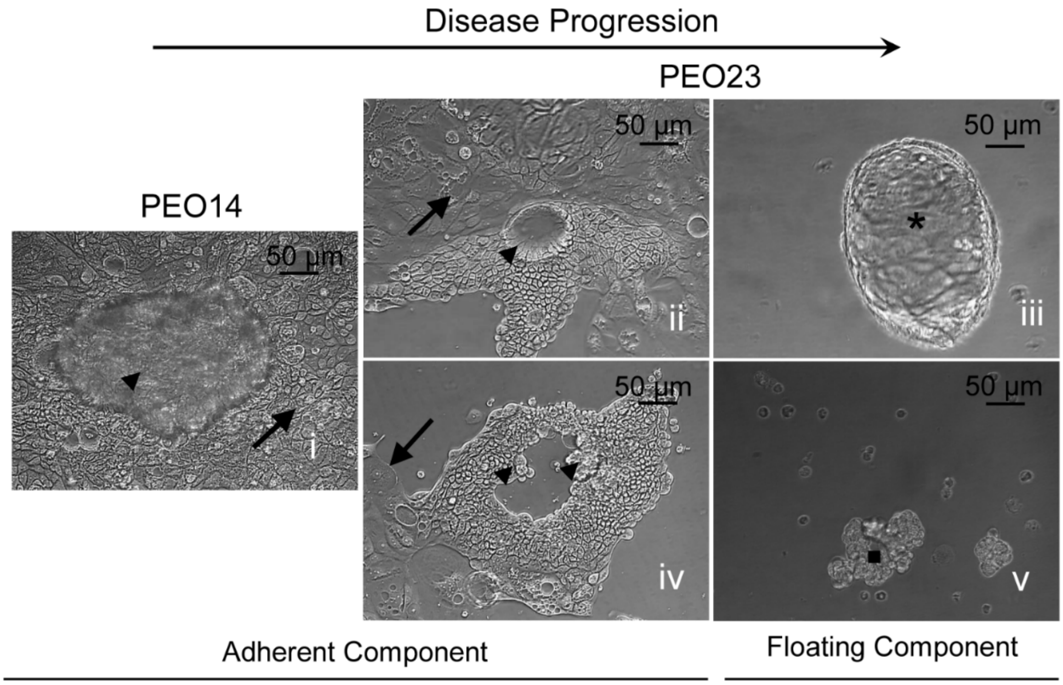

Figure S1. PEO14 and PEO23 cells depict a flat adherent component (*arrows*) and 3D foci (*arrowheads*). PEO23 foci develop multicellular structures either irregular (*square*) or spheroidal (*asterisk*).

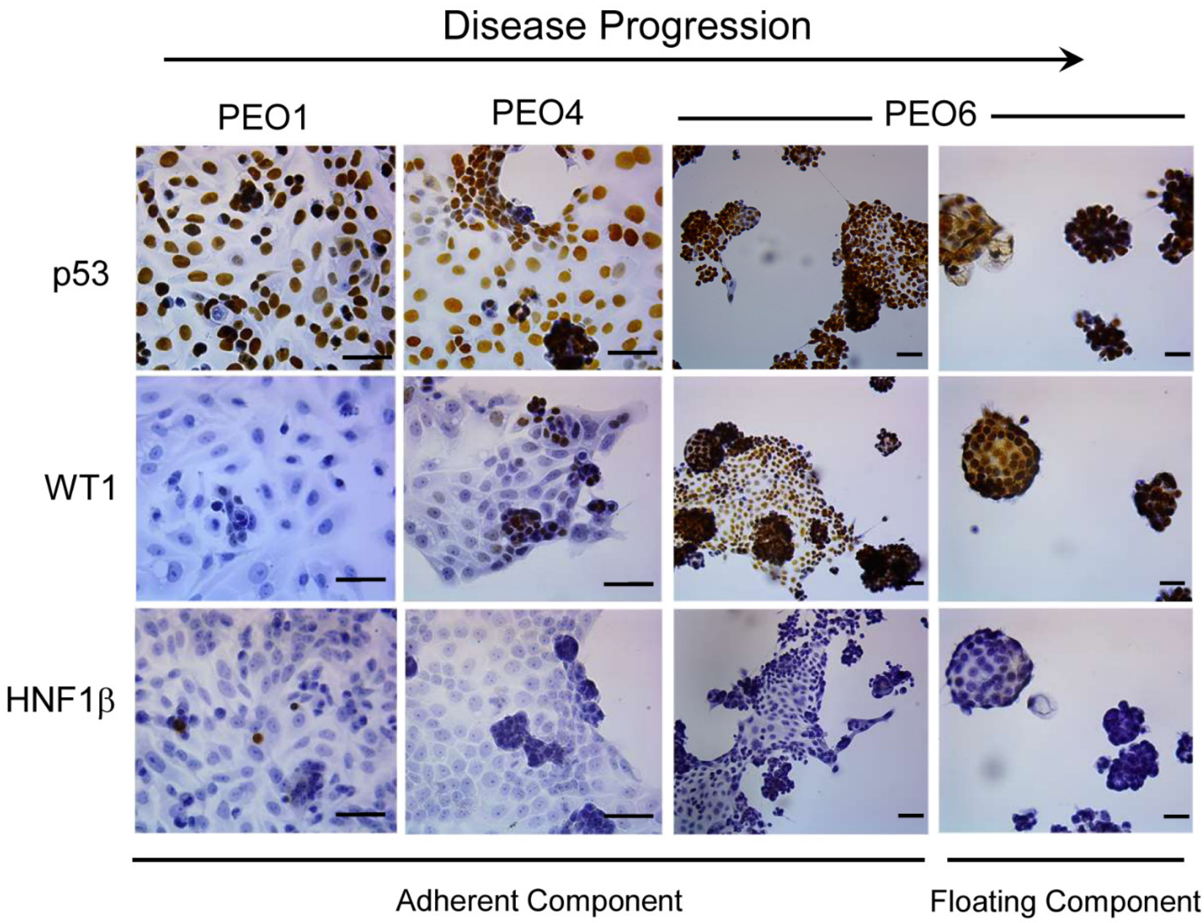

Figure S2. Multicellular structures express p53 and WT1, and are negative for the clear carcinoma cell marker HNF1β. The expression of p53 and WT1 is stronger in the foci and spheroidal structures when compared to cells growing in a monolayer. Scale bar=50 μm.

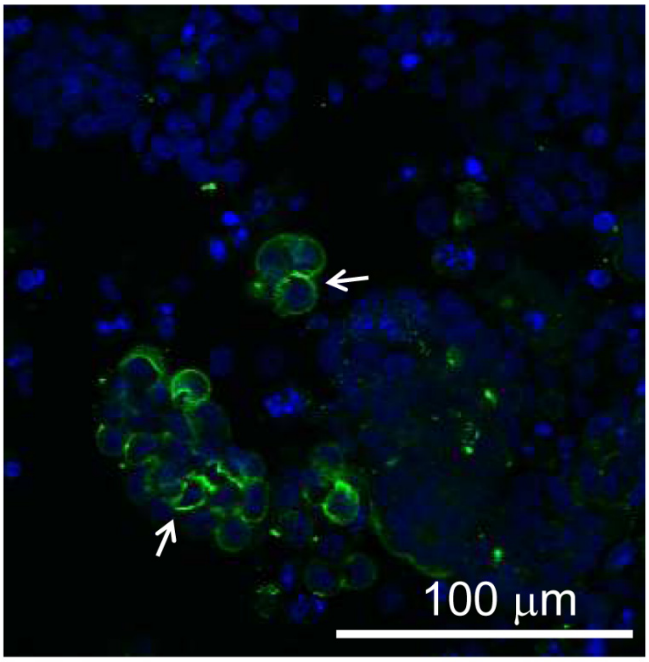

Figure S3. CD133 positive clusters of non-adherent PEO6 cells as denoted via white *arrows*.

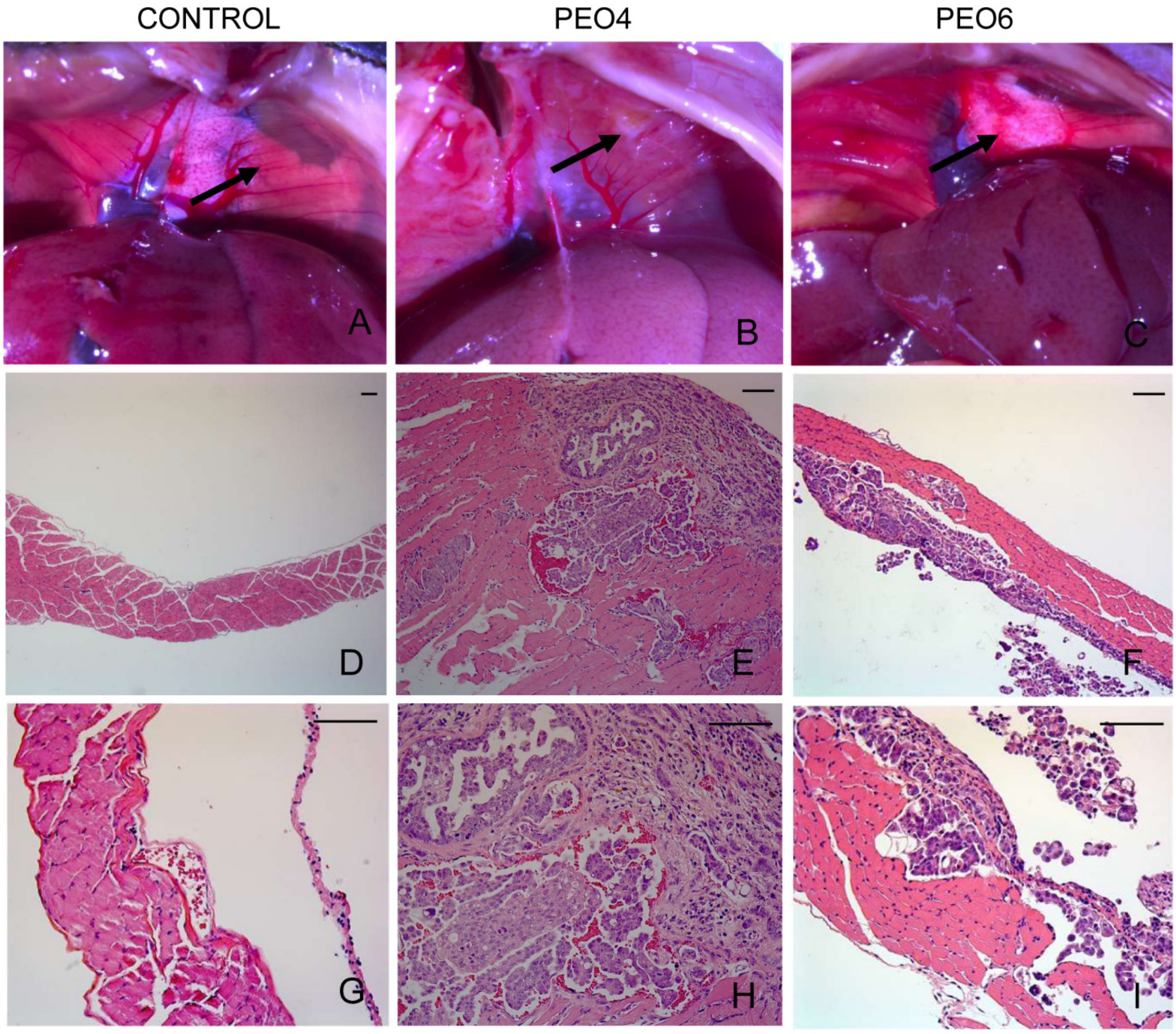

Figure S4. Images denote the presence of small foci of disease in the diaphragm of animals injected with either PEO4 or PEO6 cells [*arrows* in (**B**) and (**C**)] that are not observed in control animals [arrow in (**A**)]. Normal tissue diaphragm is shown at different magnifications (**D** and **G**). PEO4 disease is observed as a micropapillary front in (**E**) and (**H**) for PEO4 cells, and as an invasive phenotype in PEO6 cells (**F** and **I**). Scale bar=100 µm.

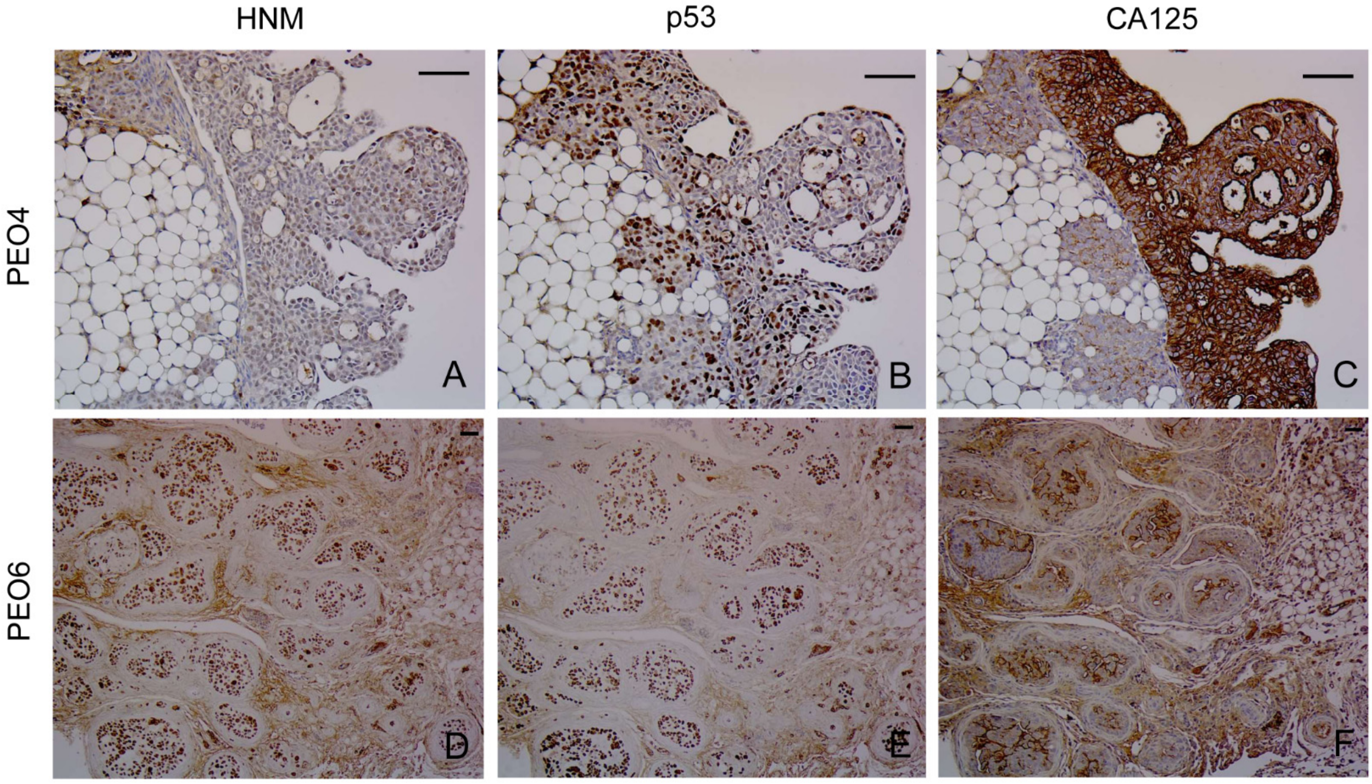

Figure S5. Metastasis taking the fat that surrounds the ovary. Positive staining of cells labeling for HNM, p53, and CA125 is shown for both PEO4 cells (**A**–**C**) and PEO6 cells (**D**–**F**). Scale bar=50 μm.

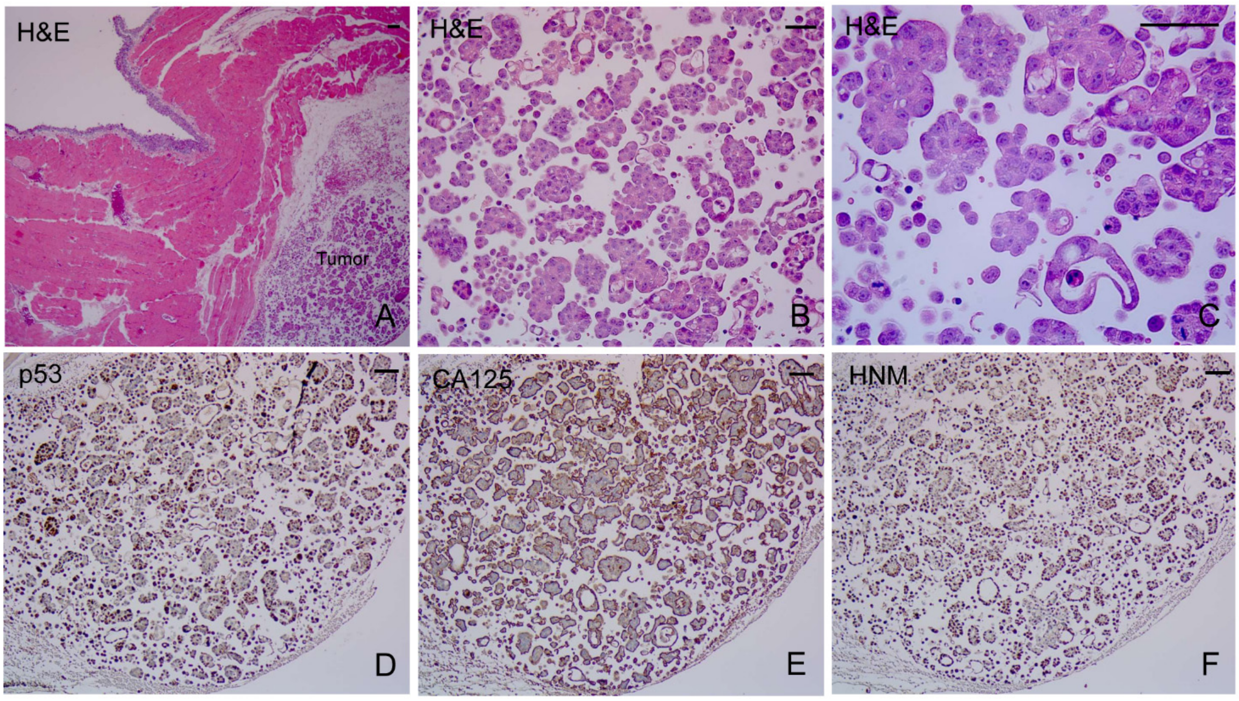

Figure S6. (**A**) PEO6 cells arranged as a tumor next to the peritoneal wall. (**B**) and (**C**) are H&E-stained areas where multicellular structures are very abundant displaying micropapillary architecture. (**D**), (**E**), and (**F**) show the positivity of such multicellular structures for p53, CA125, and HNM, respectively. Scale bar=100 µm.

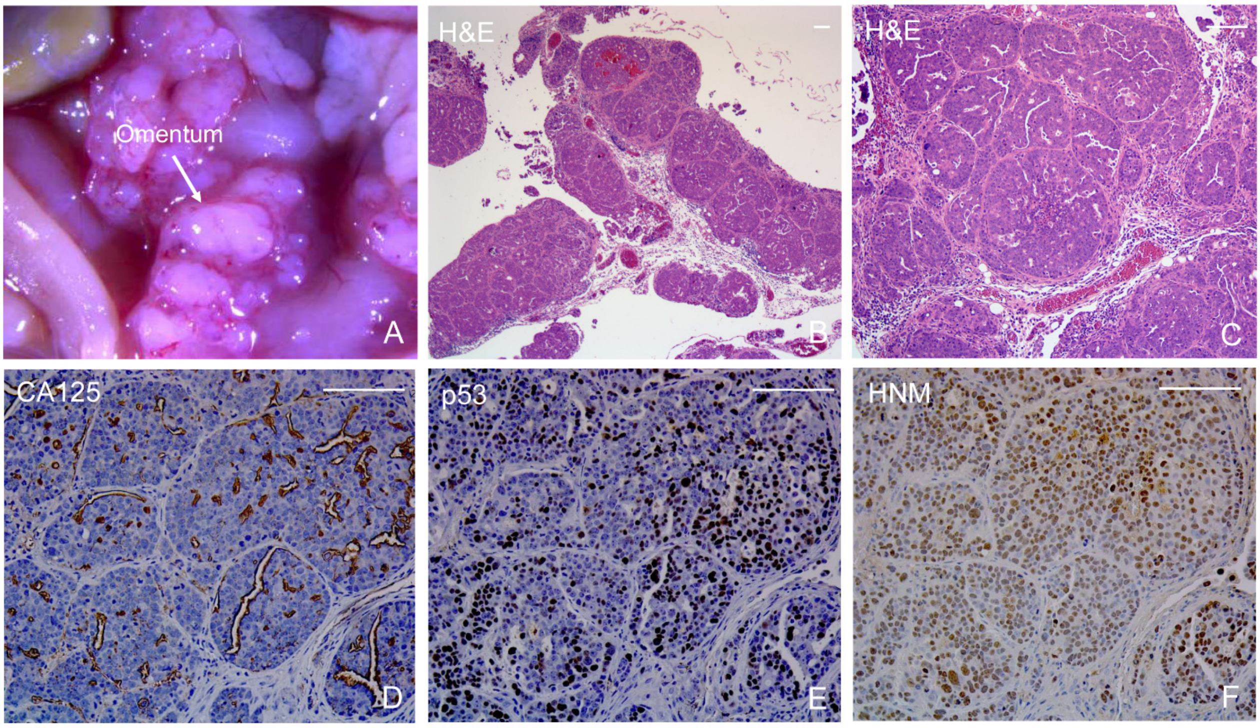

Figure S7. Macroscopic and microscopic view of omental metastasis generated when nude mice were injected with multicellular structures from PEO6 cells. (**A**) Macroscopic view. (**B**) Low magnification image denoting the omentum taken by nodules of cancerous tissue. (**C**) High magnification of tumoral section stained with H&E denoting papillary tissue with slit-like areas. (**D**) CA125; (**E**) p53; (**F**) HNM. Scale bar=100 µm.

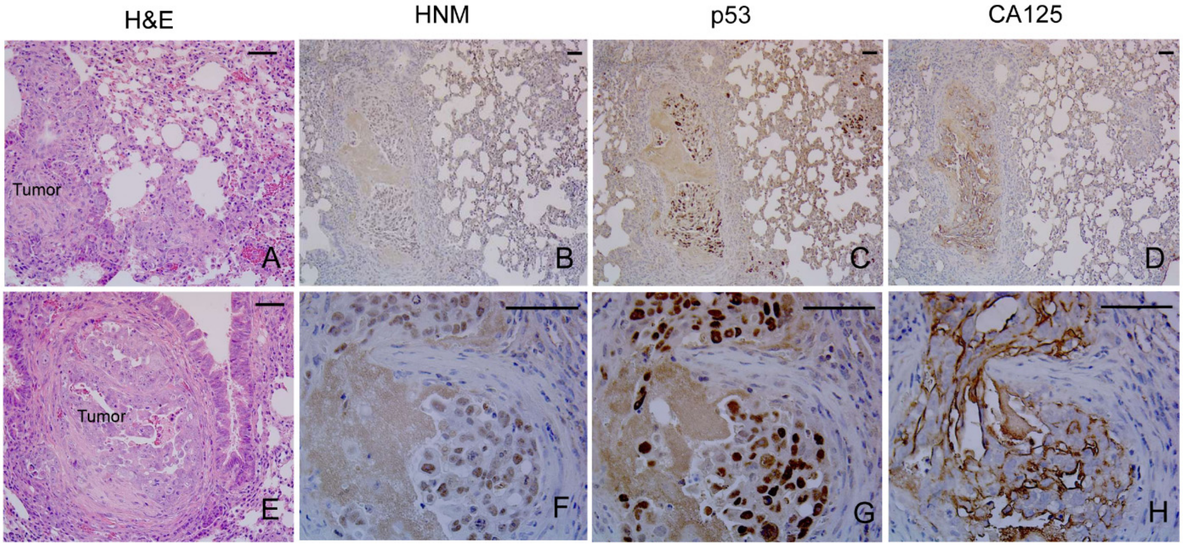

Figure S8. Lung metastasis. Images show a case of PEO6 multicellular structures capable of reaching the lung and forming a metastatic growth. The metastasis is clearly observed in H&E-stained sections at low (**A**) or high (**E**) magnifications. Low and high magnifications of the lung metastasis also are observed for HNM (**B**, **F**), p53 (**C**, **G**), and CA125 (**D**, **H**). Scale bar=50 µm.

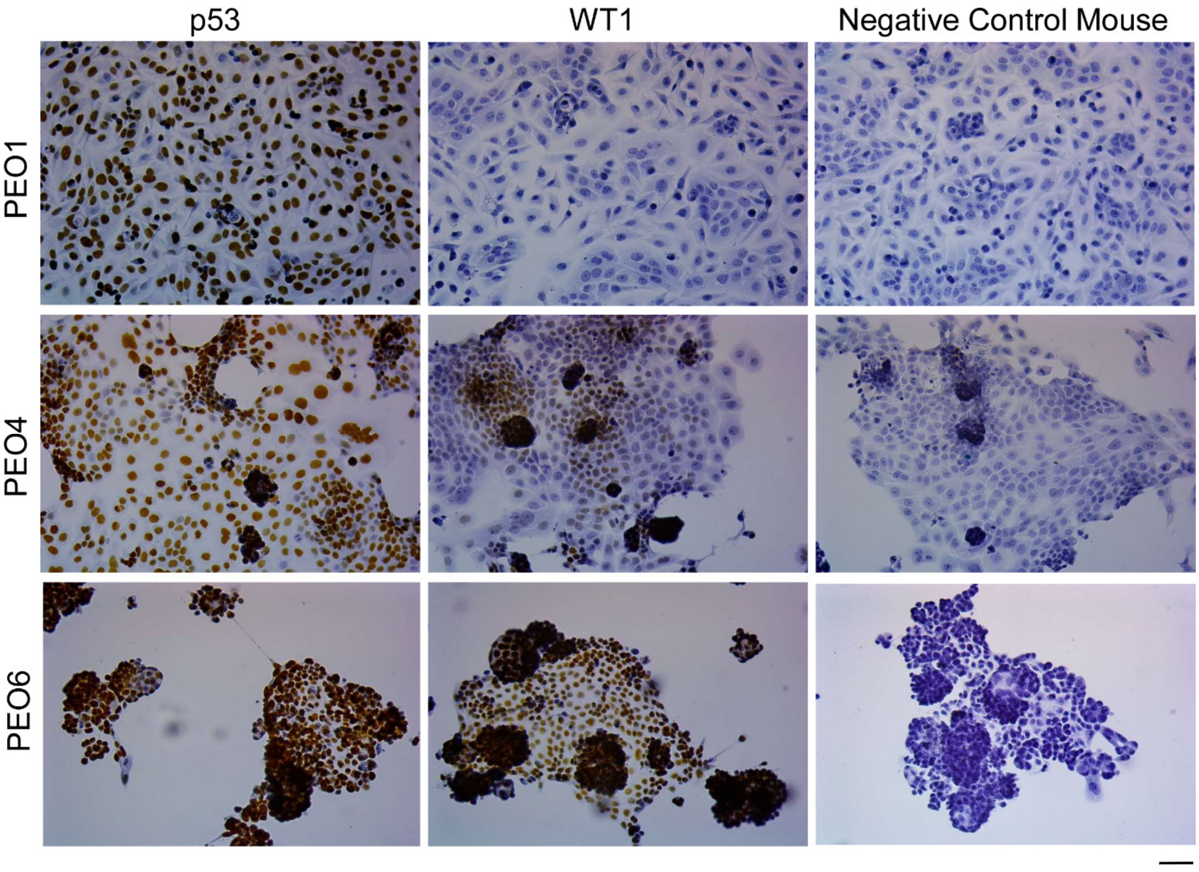

Figure S9. Negative controls for the immunostaining of WT1 and p53 in cultured PEO1, PEO4, and PEO6 cells originally presented in Figure 1B. Scale bar=50 µm.

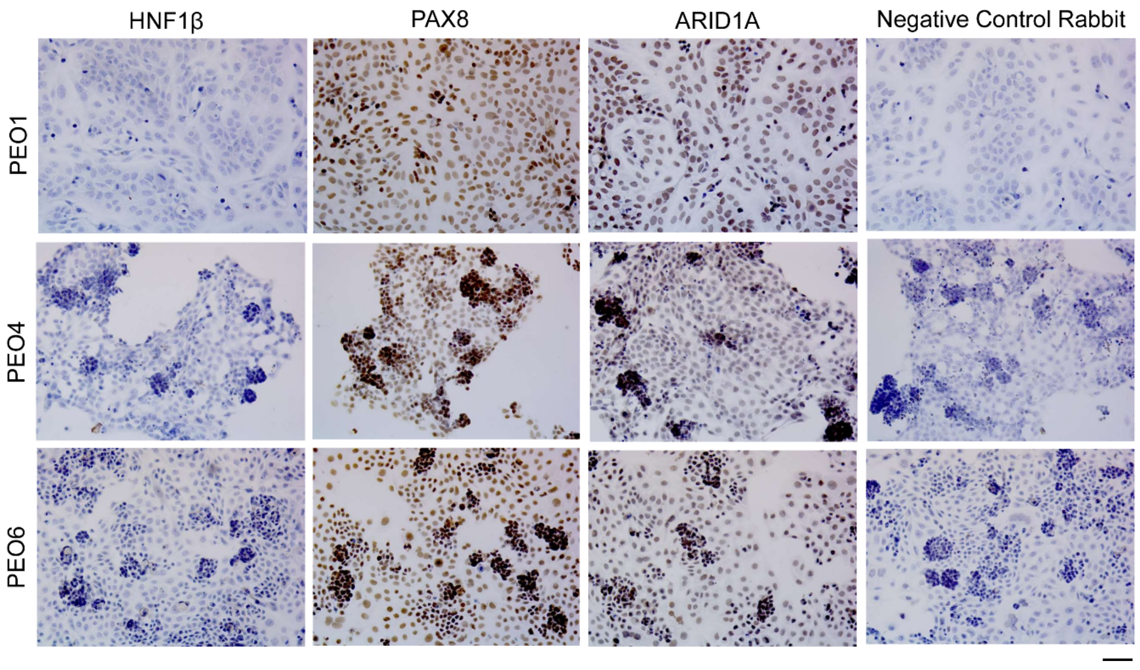

Figure S10. Negative controls for the immunostaining of HNF1β, PAX8, or ARID1A in cultured PEO1, PEO4, and PEO6 cells presented in Figure 1B. Scale bar=50 µm.

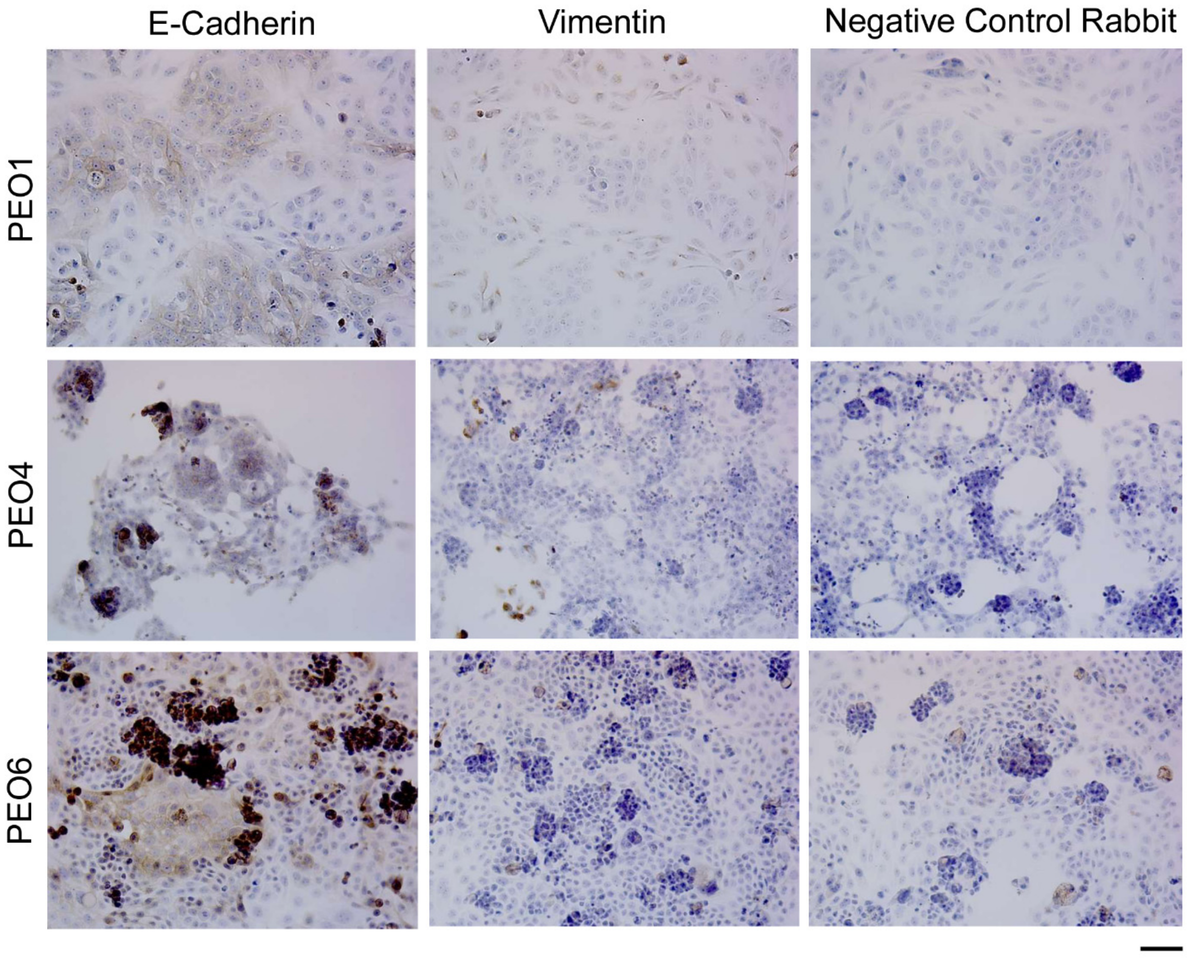

Figure S11. Negative controls for the immunostaining of E-cadherin and vimentin in cultured PEO1, PEO4, and PEO6 cells presented in Figure 1C. Scale bar=50 µm.

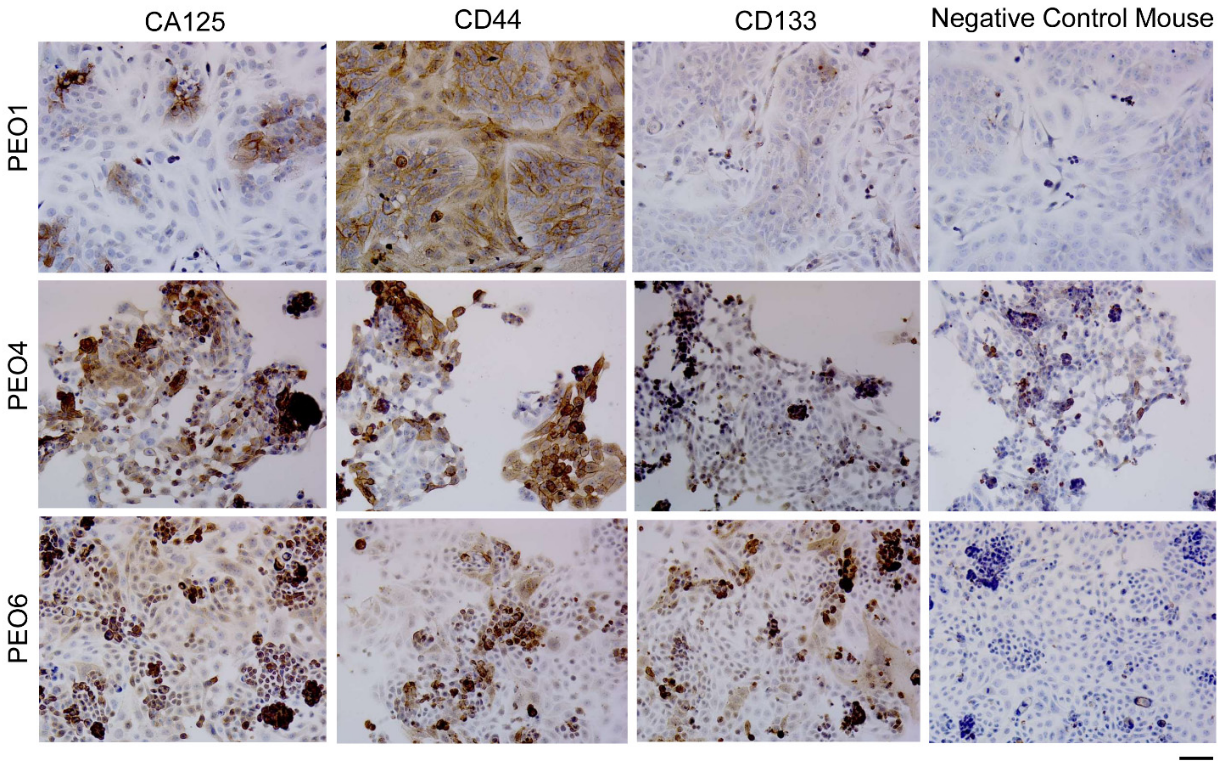

Figure S12. Negative controls for the immunostaining of CA125, CD44, and CD133 in cultured PEO1, PEO4, and PEO6 cells originally presented in Figure 1C. Scale bar=50 μm.

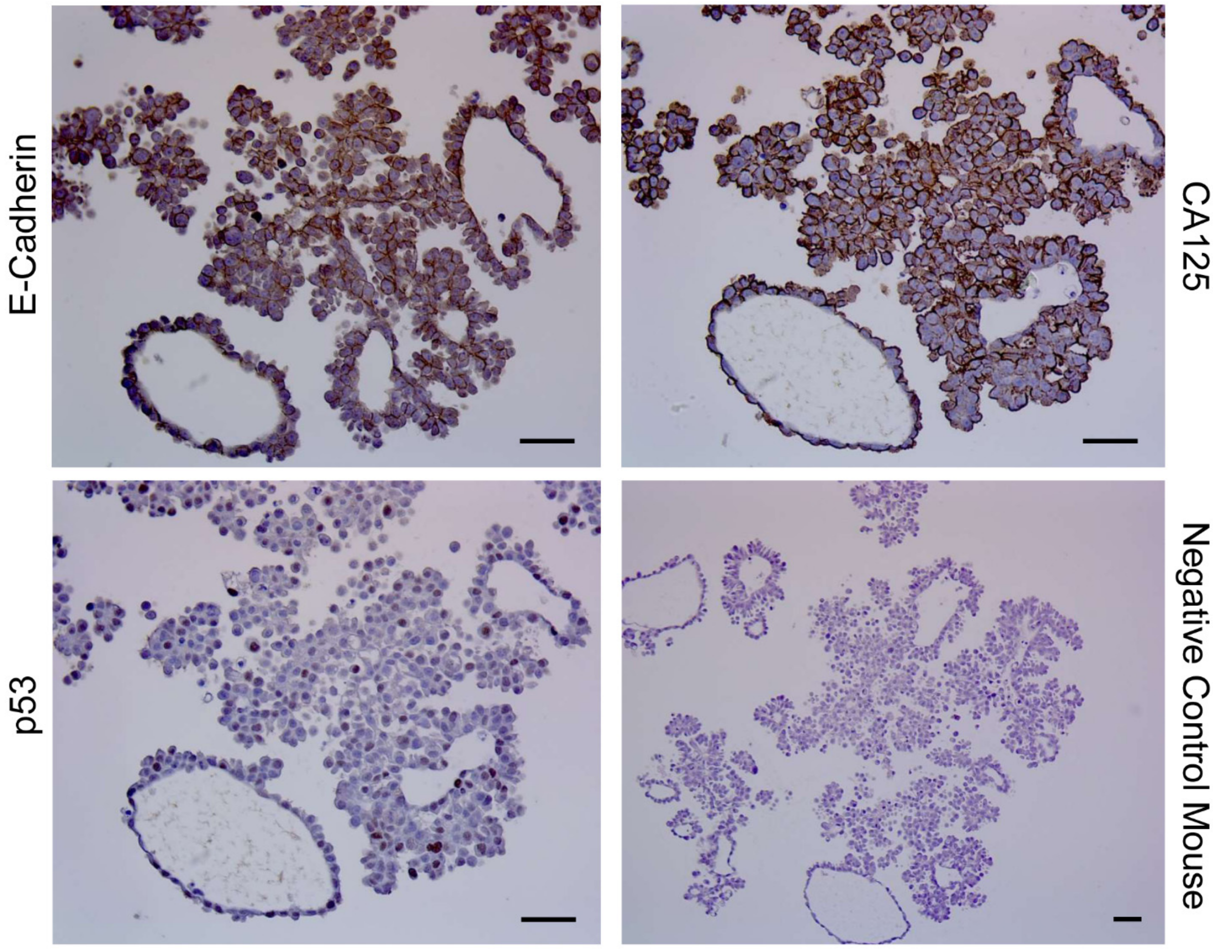

Figure S13. Negative controls for the immunostaining of CA125, E-cadherin, and p53 in cultured multicellular structures (MCS) of PEO6 cells and originally presented in Figure 3B. Scale bar=50 μm.

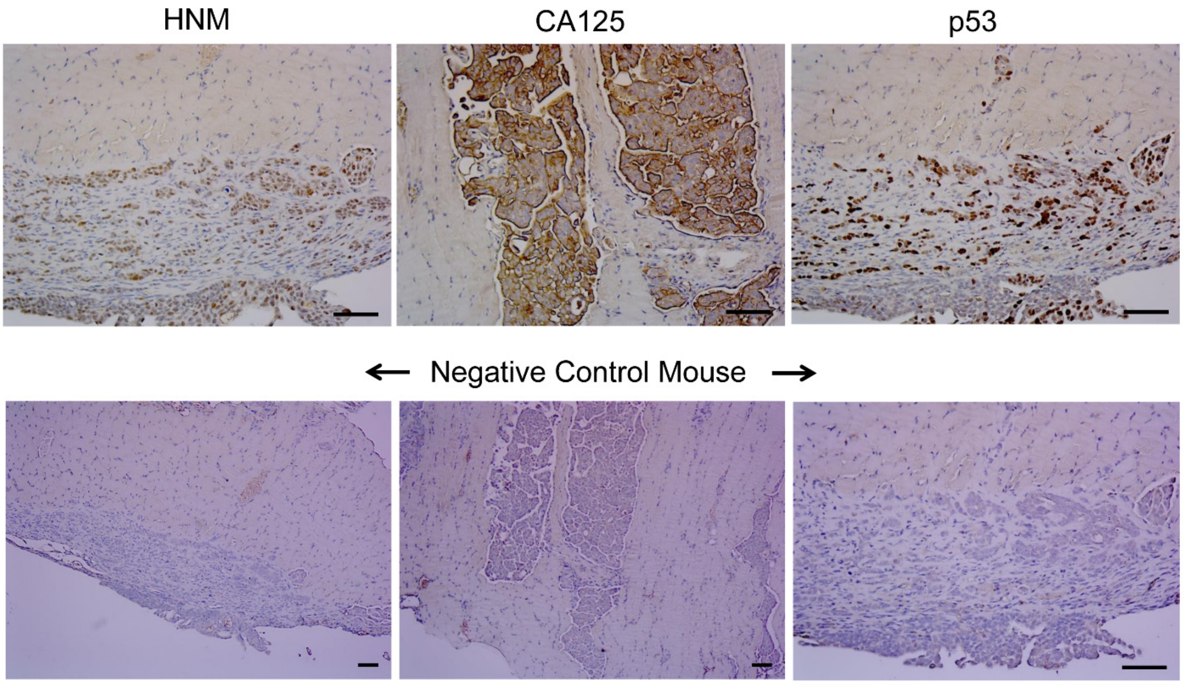

Figure S14. Representative negative controls for the immunostaining of HNM, CA125, and p53 as observed in the diaphragm of nude mice that developed metastases upon intraperitoneal administration of PEO4 cells. Scale bar=50 µm.

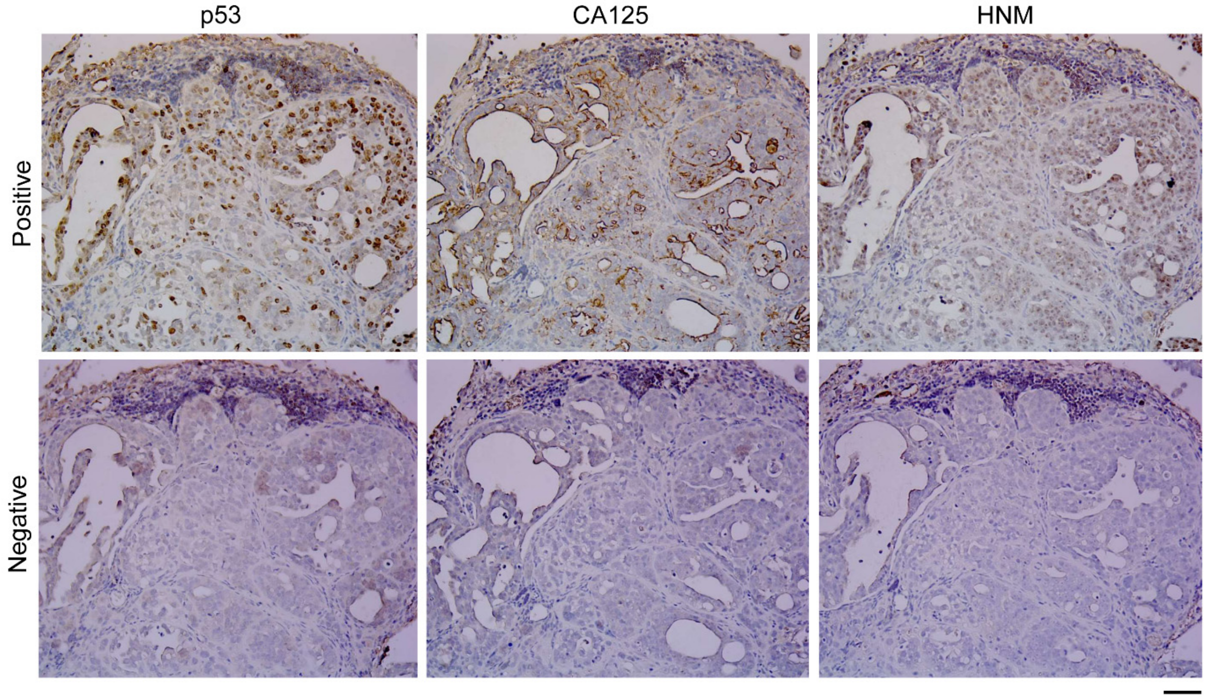

Figure S15. Representative positive immunostaining for p53, CA125, and HNM with their corresponding serial sections not incubated with the primary antibody (negative). Images correspond to the omental area of nude mice that developed metastases upon injection of PEO6 cells (compare with Figure 5B). Scale bar=50 µm.

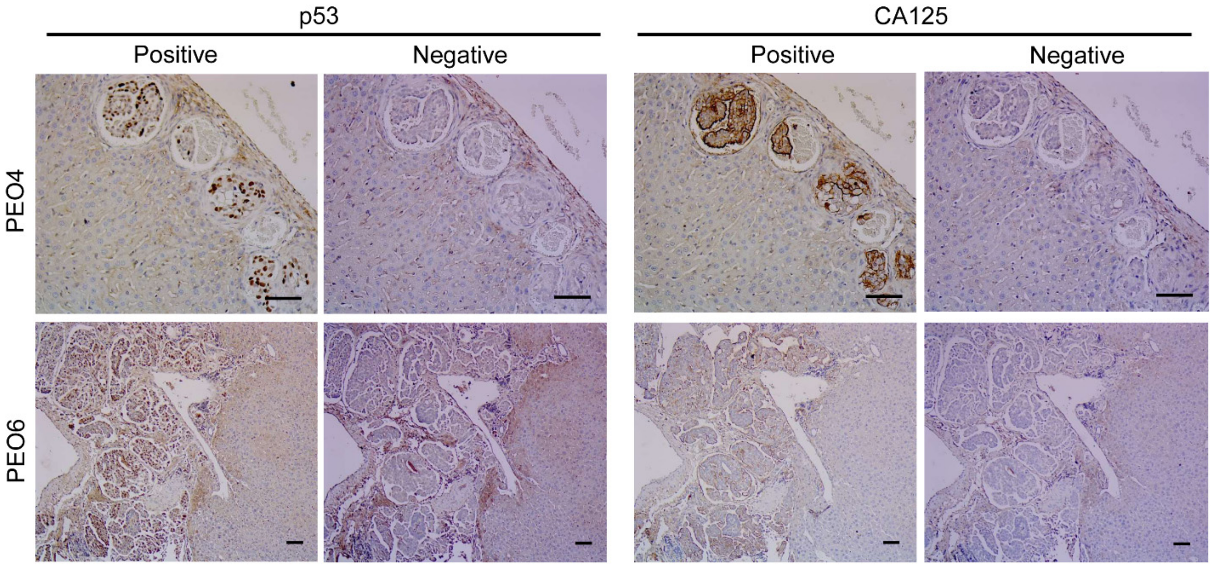

Figure S16. Representative positive immunostaining for p53 and CA125 as observed in micro-metastases found at the base of the liver of animals that were injected with either PEO4 or PEO6 cells. Negative images were generated in serial sections that were incubated in the absence of the primary antibody (negative). Scale bar=50 μm.

Table S1. Source and dilutions of antibodies utilized in this work.

Antibody	Catalogue	Company	Dilution
p53	M7001	Dako	1:400
WT-1	M3561	Dako	1:50
PAX8	10336-1-AP	Proteintech	1:1,000
HNF1β	HPA002083	Sigma Chemical Company	1:100
ARID1A	HPA005456	Sigma Chemical Company	1:75
E-cadherin	3195	Cell Signaling	1:50
Vimentin	5741	Cell Signaling	1:50
CA125	M5-1151-5	Thermo Fisher Scientific	As is
CD44	MCICD44	Novocastra Laboratories	1:100
CD133-1	130-090-4	Miltenyi Biotec	1 μg/ml
Anti-human nucleoli marker	ab190710	abcam	2 μg/ml
β-catenin	8480	Cell Signaling	1:100
ImmPress HRP anti-rabbit	MP-7401	Vector Laboratories	As is
ImmPress HRP anti-mouse	MP-7402	Vector Laboratories	As is
Goat-anti rabbit IgG, Alexa Fluor 488	A-11034	Thermo Fisher Scientific	1:1,000
